# Adenosine kinase protects against acetaminophen-induced acute liver injury by activating autophagy in hepatocytes

**DOI:** 10.1007/s10565-024-09906-0

**Published:** 2024-07-27

**Authors:** Chuanxin Zhang, Xuehao Liu, Xilong Liu, Rui Hua, Han Liu, Jiaxin Ma, Dan Zou, Guangmei Wang, Qiuhuan Yuan, Bailu Wang, Shujian Wei, Yuguo Chen

**Affiliations:** 1 Department of Emergency and Chest Pain Center, Qilu Hospital of Shandong University, Jinan, 250012 Shandong China; 2 Shandong Provincial Clinical Research Center for Emergency and Critical Care Medicine, Institute of Emergency and Critical Care Medicine of Shandong University, Qilu Hospital of Shandong University, Jinan, 250012 Shandong China; 3 Key Laboratory of Emergency and Critical Care Medicine of Shandong Province, Key Laboratory of Cardiopulmonary-Cerebral Resuscitation Research of Shandong Province, Qilu Hospital of Shandong University, Jinan, 250012 Shandong China; 4 The Key Laboratory of Cardiovascular Remodeling and Function Research, Chinese Ministry of Education, Chinese Ministry of Health and Chinese Academy of Medical Sciences, Qilu Hospital of Shandong University, Jinan, 250012 Shandong China; 5 NMPA Key Laboratory for Clinical Research and Evaluation of Innovative Drug, Clinical Trial Center, Qilu Hospital of Shandong University, Jinan, 250012 Shandong China

**Keywords:** Acute liver injury, Adenosine kinase, Autophagy, mTOR, Adenosine receptor A1

## Abstract

Acute liver injury (ALI) is a common life-threatening condition with a high mortality rate due to liver disease-related death. However, current therapeutic interventions for ALI remain ineffective, and the development of effective novel therapies is urgently needed. Liver samples from patients with drug-induced ALI were collected to detect adenosine kinase (ADK) expression. Male C57BL/6 J mice, hepatocyte-specific ADK knockout (*ADK*^*HKO*^) mice, and their controls (*ADK*^*f/f*^) were exposed to acetaminophen (APAP) and other treatments to investigate the mechanisms of APAP-related ALI. ADK expression was significantly decreased in APAP-injured livers. Hepatocyte-specific ADK deficiency exacerbated APAP-induced ALI, while a gain-of-function approach delivering AAV-ADK, markedly alleviated APAP-induced ALI, as indicated by changes in alanine aminotransferases (ALT) levels, aspartate aminotransferase (AST) levels, neutrophil infiltration and hepatocyte death. This study showed that ADK played a critical role in ALI by activating autophagy through two signaling pathways, the adenosine monophosphate-activated protein kinase (AMPK)-mTOR pathway and the adenosine receptor A1 (ADORA1)-Akt-mTOR pathway. Furthermore, we found that metformin upregulated ADK expression in hepatocytes and protected against APAP-induced ALI. These results demonstrate that ADK is critical in protecting against APAP-induced ALI and that developing therapeutics targeting ADK-adenosine-ADORA1 is a new approach for ALI treatment. Metformin is a potential candidate for preventing ALI by upregulating ADK.

**Graphical Abstract:**

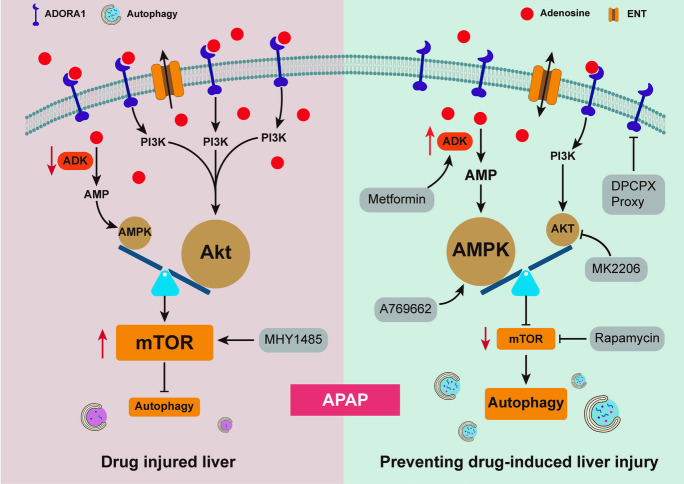

**Supplementary Information:**

The online version contains supplementary material available at 10.1007/s10565-024-09906-0.

## Introduction

Acute liver injury (ALI), which is accompanied by extensive hepatocyte death, leads to loss of liver function (liver failure) and frequently, a fatal outcome (Stravitz and Lee 2019). Acetaminophen (APAP) accounts for most ALI cases in many countries (Duan et al. 2022). APAP is widely used as a safe and effective analgesic and antipyretic drug at therapeutic doses. However, overdose can result in liver injury and potentially lead to acute liver failure (ALF) (Jaeschke and Ramachandran 2024). APAP-induced liver injury can be roughly divided into two overlapping stages. In the early stage, excessive APAP is metabolized by cytochrome P450 enzymes into the toxic compound N-acetyl-p-benzoquinone imine (NAPQI). This metabolite depletes glutathione and binds to cysteine residues on mitochondrial proteins, resulting in mitochondrial dysfunction and an overproduction of reactive oxygen species (ROS) (Heldring et al. 2022; Raith et al. 2024). In the late stage, several danger-associated molecular patterns (DAMPs) are released from necrotic hepatocytes, promoting the inflammatory response in the liver (Jaeschke and Ramachandran 2020).

The only therapeutic option for APAP-induced liver injury is N-acetyl cysteine (NAC), which detoxifies reactive oxygen species (ROS) and peroxynitrite (Walayat et al. 2021). However, most patients with APAP-induced liver injury seek medical care during or after the peak of injury, and the effect of NAC is limited at this late stage (Du et al. 2016). Thus, exploring the molecular mechanisms underlying APAP-induced liver injury to develop new drugs is important (Jaeschke et al. 2020). Recently, activation of the adenosine A2B receptor was reported to have a beneficial effect on liver recovery after APAP overdose beyond the therapeutic window of NAC (Duan et al. 2022). Adenosine receptors, including A2B receptors, are activated by extracellular adenosine. Both extracellular and intracellular adenosine levels are regulated by adenosine kinase (ADK), which metabolizes adenosine to produce adenosine monophosphate (AMP) (Wang et al. 2021). Thus, we speculate that ADK might contribute to APAP-induced liver injury.

In this study, we investigated a new therapeutical target for ALI. We found that ADK expression was downregulated in drug-injured livers. After knocking out or overexpressing ADK in mice, we found that ADK plays an important role in ALI. The underlying mechanisms through which ADK affects ALI were explored, and a new strategy for increasing ADK expression was described.

## Methods

### Human samples

Injured liver samples were collected from patients with drug-induced liver injury (DILI) who underwent liver transplantation (n = 4), and healthy controls who were liver donors (n = 4). Informed consent was obtained from each participant. The demographic characteristics of the enrolled subjects are presented in Supplemental Table 1.

### Experimental animals

Mice were housed in specific pathogen-free conditions at 22–24 °C with a 12-h light/12-h dark cycle. Male wild-type (WT) C57BL/6 J mice, aged 6–8 weeks, were obtained from Beijing Vital River Laboratory Animal Technology Co., Ltd. (Beijing, China). *ADK*^*flox/flox*^ and *Alb*-creERT2 mice were purchased from Cyagen Biosciences Inc. (Suzhou, China). *ADK*^*HKO*^ (*ADK*^*flox/flox*^-*Alb*-creERT2) mice were generated by crossing *ADK*^*flox/flox*^ mice with *Alb*-creERT2 mice. To activate Cre recombinase activity in the CreERT2 system, tamoxifen (Tm, Sigma T5648) was dissolved in corn oil (Aladdin, C116025) following the procedure described in a previous study (Reinert et al. 2012). The solution (with a concentration of 20 mg/ml) was administered intraperitoneally to *ADK*^*HKO*^ mice (40 mg/kg) for five consecutive days. For the liver-specific overexpression of ADK, AAV8-TBG-ADK-P2A or AAV8-TBG adeno-associated virus (Vigene Bioscience, Jinan, China) was used a pretreatment and administered via tail vein injection at a dose of 8*10^12^ vg/kg 30 days before APAP challenge.

### Mouse treatment

A drug-induced hepatotoxicity model was constructed by administering 300 mg/kg APAP (Selleck, S1634) intraperitoneally to mice after an overnight fast and treating them continuously for 24 h. Other APAP exposure doses and times may be used depending on specific experimental needs. In addition, the details of the small-molecule agonists and inhibitors administered in vivo are shown in Supplemental Table 2.

### Cell culture

The AML12 cell line (CL-0602) was provided by Procell Life Science & Technology Co., Ltd. (Wuhan, China). The cells were cultured under permissive conditions (37 °C, 5% CO_2_) in DMEM/F-12 (Gibco, #C11330500BT) supplemented with 10% fetal bovine serum (FBS) and 1% penicillin–streptomycin. Primary hepatocytes and nonparenchymal cells (NPCs) were isolated by in situ liver perfusion as described previously (Liu et al. 2020). Mouse primary hepatocytes were cultured in William’s E medium (Gibco, 12551032) solution containing 10% FBS and 1% penicillin–streptomycin at 37 °C with 5% CO2 on collagen I-coated plates. NPCs were cultured in DMEM (Gibco, C11995500) medium containing 10% FBS and 1% penicillin–streptomycin. The above cell lines were challenged with APAP (10 mM) for 24 h. Unless otherwise specified, the following concentrations of reagents, all purchased from Selleck, were applied in vitro: MHY1485 (10 μM), DPCPX (0.5 μM), ABT702 (1 μM), CCPA (10 μM), and metformin (5 mM). All the reagents were administered 2 h before the APAP challenge.

### Biochemical analysis

Serum alanine aminotransferase (ALT) and serum glutamic oxaloacetic transaminase (AST) levels were measured by BS-240 VET blood chemistry analyzer (Mindray, Shenzhen, China) following the manufacturer's instructions.

### Histological staining

Liver sections were stained with hematoxylin and eosin (H&E). Cell apoptosis was assessed by TUNEL staining of liver sections using a commercial kit (MK1015, Boster, Wuhan, China). Cell death was calculated by the ratio of positive cells to the total number of cells in the field of view. Immunohistochemical analysis was conducted on 3-μm-thick formalin-fixed and paraffin-embedded liver samples. The antibodies used for immunohistochemistry are as follows: anti-ADK (1:200, Abcam), anti-MPO (1:200, Proteintech), and anti-adenosine Receptor A1 (ADORA1) (1:200, Proteintech). The staining procedure was performed according to previously described methods (Zhao et al. 2019). Using ImageJ software, the positive expression area of the target protein is calculated and then divided into the total area of a 10 × magnification field of view. Multiplex immunohistochemistry (mIHC) was performed on 3-μm-thick formalin-fixed, paraffin-embedded whole tissue sections using a Quadruple-Fluorescence Immunohistochemical Mouse/Rabbit Kit (RS0036, Immunoway) for sequential pairing of standard primary antibodies, followed by DAPI staining. The primary antibodies employed were: anti-ADK (1:200, Abcam) and anti-HNF4α (1:200, ABclonal). For immunofluorescence, frozen liver Sects. (6 μm) were incubated with anti-LC3b (1:200, Abcam), anti-LAMP1 (1:100, CST), and DAPI (Abcam). Stained liver tissues were visualized with an automated fluorescence microscope (Olympus BX63).

### Western blot analysis

The extracted proteins were quantified as previously described (Zhao et al. 2019). Protein samples underwent separation by 8% or 12% sodium dodecyl sulfate–polyacrylamide gel electrophoresis (SDS-PAGE) and were subsequently transferred to 0.22 μm polyvinylidene difluoride (PVDF) membranes (Merck Millipore, Billerica, MA). Following a 1.5-h blocking period at room temperature with 5% nonfat milk in TBS containing 0.1% Tween-20 (TBST), the membranes were then incubated overnight at 4 °C with primary antibodies. The primary antibodies used are detailed in Supplemental Table 3. Protein bands were detected using an enhanced chemiluminescence reagent (Merck Millipore, WBKLS0500) in conjunction with horseradish peroxidase-conjugated secondary antibodies. Band quantification was carried out using ImageJ software (version 1.53c, NIH).

### Measurement of Glutathione and Oxidized Glutathione

Glutathione (GSH) and oxidized glutathione (GSSG) levels were determined using a micro-reduced GSH test kit (BC1175, Solarbio) and a GSSG content assay kit (BC1185, Solarbio), respectively. Briefly, the levels of reduced GSH and GSSG were measured by a spectrophotometer at 412 nm.

### RNA sequencing (RNA-seq) analysis

The RNA libraries were sequenced on the Illumina NovaSeqTM 6000 platform by LC Biotechnology Co., Ltd. (Hangzhou, China). After generating the final transcriptome, String Tie and Ballgown (Kovaka et al. 2019; Pertea et al. 2015) were utilized to estimate transcript expression levels and determine mRNA abundance by calculating fragments per kilobase of transcript per million mapped reads (FPKM) values. Bioinformatic analysis was conducted utilizing the OmicStudio tool at https://www.omicstudio.cn/tool. Differential expressed genes (DEGs) between two groups were performed using DESeq2 software, while edgeR was employed for comparison between two samples. Genes with a false discovery rate (FDR) < 0.05 and |log_10_FC|≥ 1.5 were considered DEGs. These DEGs underwent GO function and KEGG pathway enrichment analyses.

The APAP-induced ALI datasets GSE110787 and GSE111828 were retrieved from the Gene Expression Omnibus (GEO) database (https://www.ncbi.nlm.nih.gov/gds). The heatmaps were generated using the “pheatmap” package of R software (version 3.6.3).

### Autophagic flux assay

AML12 cells were transfected with the recombinant adenovirus mRFP-GFP-LC3 (Vigene Bioscience, Jinan, China). Autophagic flux was measured by assessing the expression of LC3 fluorescent fusion proteins, which included yellow (autophagosomes) and red (autolysosomes) puncta, under a confocal microscope (Leica SP8).

### Transmission *electron* microscopy (TEM)

The fresh liver tissue samples were fixed with 2.5% glutaraldehyde for two hours and subsequently stored at 4 °C. Images were acquired using TEM (HT7700, Hitachi, Japan). Two types of autophagic vacuoles (AVs) were quantified in a high-power field (covering 100 μm^2^): autophagosomes (double membrane, no ribosomes, density -similar to cytosol) and autolysosomes (single membrane, much lower luminal density, and containing light or dense amorphous material) (n = 5 mice per group).

### Statistical analysis

Statistical analysis was conducted using GraphPad Prism 8. The experimental data are presented as the mean ± SEM. For comparisons between two groups, a two-tailed Student’s t-test was used for normally distributed data, while the Mann–Whitney U test was applied for skewed distributions. Two-way ANOVA with Bonferroni test was used for multiple comparisons with two independent variables. For comparisons among more than two groups, one-way ANOVA with Tukey test was generally employed. Survival analysis utilized the log-rank (Mantel-Cox) test.

## Results

### ADK expression is downregulated in APAP-injured livers and hepatocytes

**Fig. 1:**
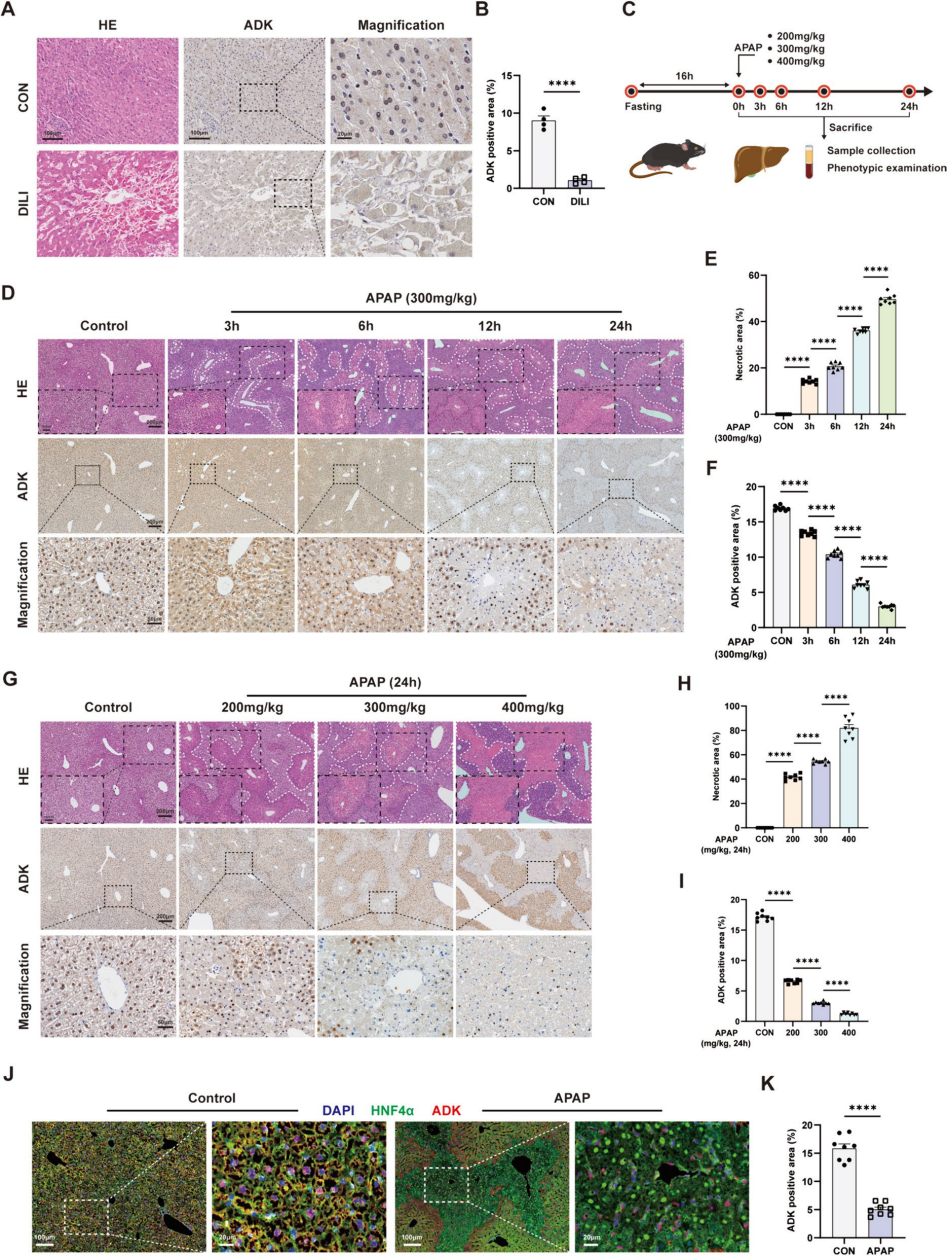
ADK expression is downregulated in APAP-injured livers or hepatocytes. (**A**-**B**) Representative immunohistochemical staining images and quantification of ADK levels in liver sections obtained from control and DILI patients (both n = 4). (**C**) Schematic diagram of APAP administration. In D-F, WT mice were intraperitoneally injected with vehicle or APAP solution (300 mg/kg). Blood and liver tissues were obtained from the mice at 0, 3, 6, 12, and 24 h (n = 8 per group). (**D**-**F**) Representative histological staining images and quantification of H&E (necrotic areas circled with white lines) and ADK staining. In G-I, WT mice were intraperitoneally injected with vehicle or various doses of APAP solution (200, 300, or 400 mg/kg). Then, the mice were sacrificed at 24 h, and liver tissues were obtained for further analysis (n = 8 per group). (**G**-**I**) Representative histological staining images and quantification of H&E and ADK staining. (**J**-**K**) Representative images and quantification of ADK on hepatocytes (HNF4α +) by mIHC in liver sections from WT mice treated with vehicle or APAP for 24 h (n = 8 per group). ^****^P < 0.0001 (Student’s *t test*, one-way ANOVA with Tukey’s test)

To investigate the potential involvement of ADK in drug-related ALI, liver sections from patients were immunohistochemically analyzed for ADK expression and we found that there was less ADK immunostaining in drug-injured livers than in control livers (Fig. 1A and B). By searching GEO datasets, we observed decreased ADK mRNA expression in APAP-injured mouse livers. (Supplemental Fig. 1A). To confirm the changes in ADK expression, we established an APAP-induced ALI mouse model with different APAP treatment times and with different APAP doses (Fig. 1C; Supplemental Fig. 1B-1E). As expected, APAP-induced liver necrosis was observed in the centrilobular area (Fig. 1D and G). The severity of APAP-induced ALI, which was indicated by the necrotic area, was positively related to the duration of exposure and the dose (Fig. 1E and 1H). Immunochemical staining and western blotting demonstrated that APAP decreased ADK expression in a manner dependent on both time and dose. (Fig. 1D, F, G, I; Supplemental Fig. 1F-I). mIHC and immunofluorescence staining indicated that ADK in hepatocytes was obviously downregulated in APAP-injured livers (Fig. 1J and K; Supplemental Fig. 1J). Next, primary mouse hepatocytes were isolated and treated with APAP. Consistent with the findings in APAP-injured mouse livers, ADK expression was decreased in APAP-treated primary mouse hepatocytes (Supplemental Fig. 1K and L). Similar changes in ADK expression were found in APAP-treated AML12 cells (Supplemental Fig. 1M and N).

### Knockout of ADK in hepatocytes exacerbates APAP-induced ALI

**Fig.2:**
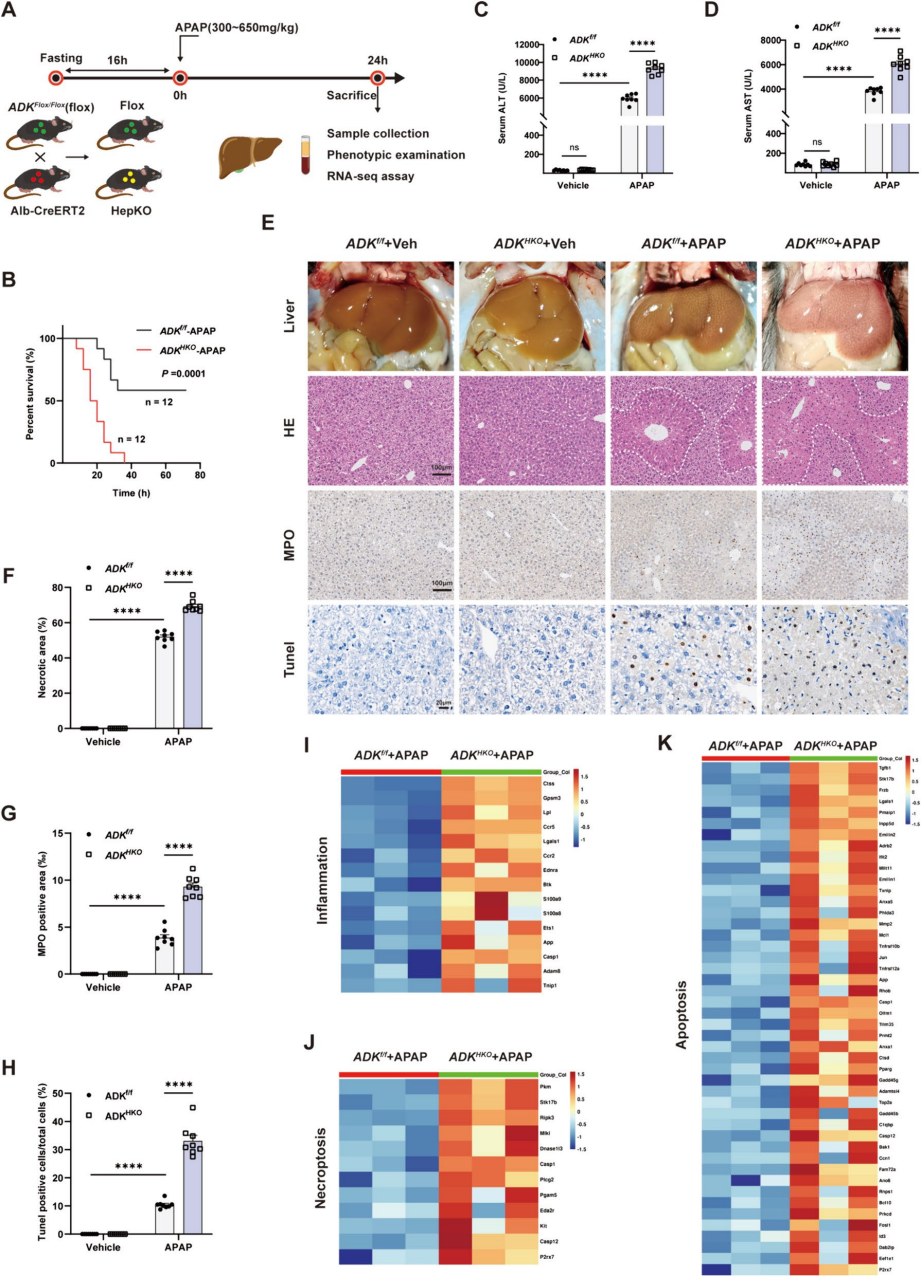
Knockout of ADK in hepatocytes exacerbates APAP-induced liver injury. (**A**) Schematic diagram of APAP administration. (**B**) Survival curves of *ADK*^*f/f*^ and *ADK*^*HKO*^ mice treated with a lethal dose of APAP (650 mg/kg) (both n = 12). In **C**-**K**, *ADK*^*f/f*^ and *ADK*^*HKO*^ mice were intraperitoneally injected with saline or APAP solution (300 mg/kg) for 24 h (n = 8 per group). (**C**-**D**) Serum ALT and AST levels. (**E**–**H**) Representative gross liver photographs and intrahepatic staining images with quantification of H&E (**F**), MPO (**G**), and TUNEL (**H**) staining. (**I**-**K**) Heatmaps showing the intrahepatic expression profiles of genes related to inflammation, necroptosis and apoptosis based on the RNA-seq dataset (both n = 3). ^****^P < 0.0001; ns, nonsignificant (two-way ANOVA with Bonferroni’s multiple comparisons test, log-rank test)

*ADK*^*HKO*^ mice were employed to study the impact of ADK in APAP-induced hepatotoxicity (Fig. 2A; Supplemental Fig. 2). When a lethal dose (650 mg/kg) of APAP was administered, hepatic ADK-deficient mice had a significantly lower survival rate than control mice (Fig. 2B). Mice were treated with APAP (300 mg/kg) for 24 h. Compared to those *ADK*^*f/f*^ mice, the serum ALT and AST levels were greater in APAP-injured *ADK*^*HKO*^ mice (Fig. 2C and D). Histological analysis showed increased liver necrosis, neutrophil infiltration and hepatocyte death in APAP-injured *ADK*^*HKO*^ mice compared to *ADK*^*f/f*^ mice (Fig. 2E-H). RNA sequencing analysis indicated enrichment of genes associated with inflammation, necroptosis, and apoptosis-related pathways was markedly greater in APAP-injured *ADK*^*HKO*^ mice compared to *ADK*^*f/f*^ mice (Fig. 2I-K). These findings indicated that hepatic ADK deficiency exacerbates APAP-related liver injury by inducing more hepatocyte death and increasing the inflammatory response.

### Autophagy was impaired by ADK knockout in APAP-injured mouse livers

**Fig. 3:**
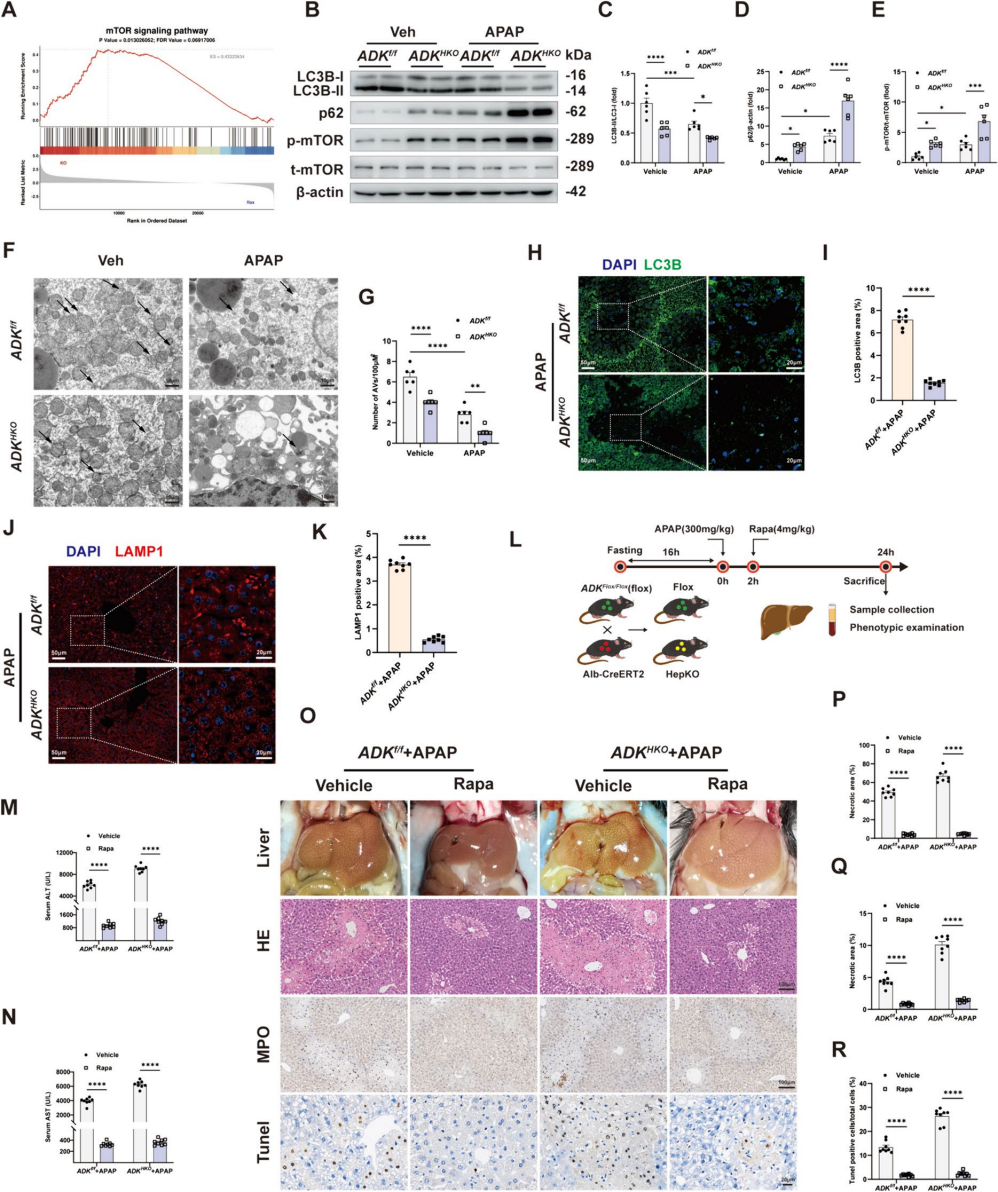
Autophagy was impaired by ADK knockout in APAP-injured mouse livers. In **A**‒**K**, *ADK*^*f/f*^ and *ADK*^*HKO*^ mice were intraperitoneally injected with saline or APAP solution (300 mg/kg) for 24 h. (**A**) GSEA showing the enriched pathways related to mTOR signaling based on the RNA-seq dataset. (**B**-**E**) Representative immunoblots showing autophagy marker quantification (n = 6). (**F**-**G**) Representative liver TEM images and quantification of AVs (black arrows, n = 6). (**H**–**K**) Representative immunofluorescence staining images and quantification of LC3B (H-I, n = 8 per group) and LAMP1 (**J**-**K**, n = 8 per group) expression. In **L**-**R**, *ADK*^*f/f*^ and *ADK*^*HKO*^ mice were treated with Rapa (4 mg/kg) after APAP challenge (n = 8 per group). (**L**) Schematic diagram of APAP and Rapa administration. (**M**–**N**) Serum ALT and AST levels. (**P**-**S**) Representative gross liver photographs and intrahepatic staining images with quantification of H&E (**P**), MPO (**Q**), and TUNEL (**R**) staining.^*^P < 0.05; ^**^P < 0.01; ^****^P < 0.0001; ns, nonsignificant (two-way ANOVA with Bonferroni’s multiple comparisons test)s

APAP is metabolized into toxic NAPQI mainly by the cytochrome P450 isozymes CYP1A2 and CYP2E1, after which NAPQI is detoxified by rapid reaction with glutathione (Zhang et al. 2018). However, knockout of ADK did not affect the expression of CYP1A2 or CYP2E1 or the level of glutathione in APAP-injured livers (Supplemental Fig. 3A-H). Autophagy plays a critical role in protecting against APAP-induced ALI (Ni et al. 2012). To determine whether autophagy mediates the effect of ADK on APAP-induced hepatotoxicity (300 mg/kg, 24 h), GSEA was performed using RNA-sequencing data. The mTOR pathway was found to be activated in APAP-injured livers from *ADK*^*HKO*^ mice compared with those from control mice (Fig. 3A). Moreover, in APAP-injured livers from *ADK*^*HKO*^ mice, the LC3B II/I ratio was reduced, but the expression of p62 and phosphorylated mTOR were increased (Fig. 3B-E). TEM analysis also demonstrated that the autophagic vacuoles was decreased in APAP-injured *ADK*^*HKO*^ livers (Fig. 3F and G). Furthermore, lower levels of LC3B and LAMP1 were detected in APAP-injured livers from *ADK*^*HKO*^ mice (Fig. 3H-K). In vitro, ADK inhibition also diminished autophagy in APAP-treated AML12 cells (Supplemental Fig. 3I-K). These results demonstrated that autophagy is impaired by hepatic ADK deficiency. To clarify the essential role of autophagy in the effects of ADK deficiency, rapamycin (4 mg/kg), an autophagy activator, was administered after APAP administration (Fig. 3L). We found that rapamycin effectively alleviated liver necrosis, neutrophil infiltration and hepatocyte cell death in the livers from APAP-treated *ADK*^*HKO*^ mice (Fig. 3M-R).

### ADK overexpression alleviates APAP-induced ALI by enhancing autophagy

**Fig. 4:**
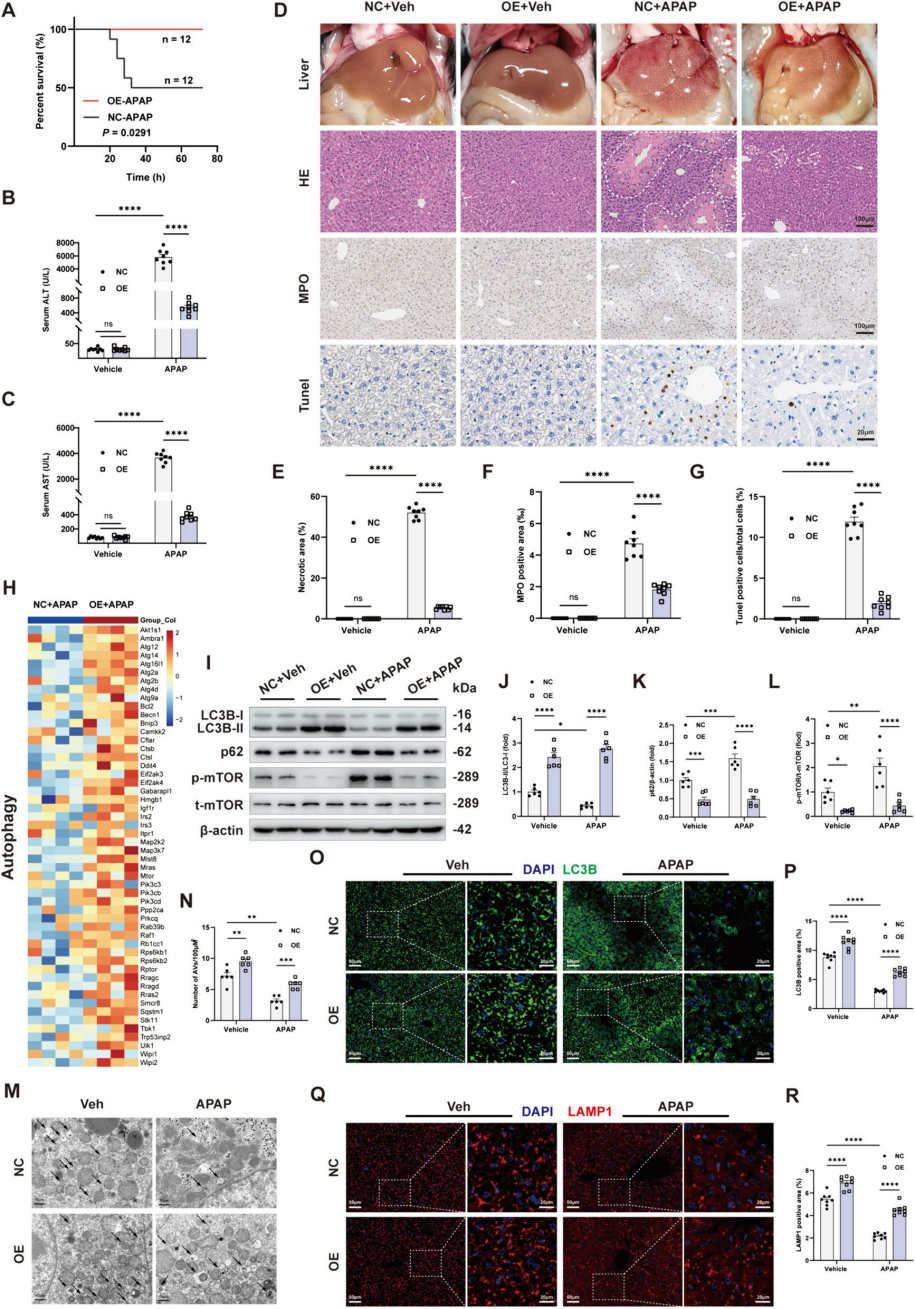
ADK overexpression alleviates APAP-induced liver injury by enhancing autophagy. In **A-R**, WT mice were injected with AAV8-TBG-ADK-P2A or AAV8-TBG adeno-associated virus 30 days before APAP challenge. (**A**) Survival curves of liver-specific ADK-overexpressing mice treated with a lethal dose of APAP (650 mg/kg) (both n = 12). In **B‒R**, mice were intraperitoneally injected with saline or APAP solution (300 mg/kg) for 24 h. (**B**-**C**) Serum ALT and AST levels (n = 8 per group). (**D**-**G**) Representative gross liver photographs and intrahepatic staining images with quantification of H&E (**E**), MPO (**F**), and TUNEL (**G**) staining (n = 8 per group). (**H**) Heatmaps showing the intrahepatic expression profiles of genes related to autophagy based on the RNA-seq dataset (n = 4). (**I**-**L**) Representative immunoblots showing autophagy marker quantification (n = 6 per group). (**M**–**N**) Representative liver TEM images showing the quantification of AVs (black arrows, n = 6). (**O**-**R**) Representative immunofluorescence staining images and quantification of LC3B (**O**-**P**, n = 8 per group) and LAMP1 (**Q**-**R**, n = 8 per group) expression. ^**^P < 0.01; ^***^P < 0.001; ^****^P < 0.0001; ns, nonsignificant (two-way ANOVA with Bonferroni’s multiple comparisons test, log-rank test)

To validate the protective role of ADK in APAP-induced liver injury, we generated ADK-overexpressing mice through adenoviral delivery via intravenous injection. (Supplemental Fig. 4A-C). When a lethal dose (650 mg/kg) of APAP was administered, hepatic ADK-overexpressing mice exhibited significant tolerance to APAP injury, and none of these mice died (Fig. 4A). Lower levels of serum ALT and AST were detected in ADK-overexpressing mice exposed to APAP (300 mg/kg, 24 h) than in control mice (Fig. 4B and C). Moreover, liver necrosis, neutrophil infiltration and cell death were alleviated by ADK overexpression in APAP-injured livers (Fig. 4D-G). Transcriptomic KEGG enrichment analysis revealed that autophagy-related pathways were strongly activated in the livers from ADK-overexpressing mice (Fig. 4H). ADK overexpression increased the LC3B II/I ratio and the phosphorylated AMPK level but decreased the expression of p62 and phosphorylated of mTOR (Fig.4I-L). TEM revealed an increased number of autophagic vacuoles in the livers from the ADK-overexpressing mice (Fig. 4M and N). Immunofluorescence staining showed increased levels of LC3B and LAMP1 in APAP-injured livers from ADK-overexpressing mice (Fig. 4O-R). Interestingly, activating mTOR prevented the protective effects of ADK overexpressing on APAP-injured livers (Supplemental Fig. 4D-J). These results indicated that autophagy activation mediates the role of ADK overexpression in protecting the liver from APAP injury.

### AMPK inactivation contributes to impaired autophagy in hepatic ADK-deficient livers

**Fig. 5:**
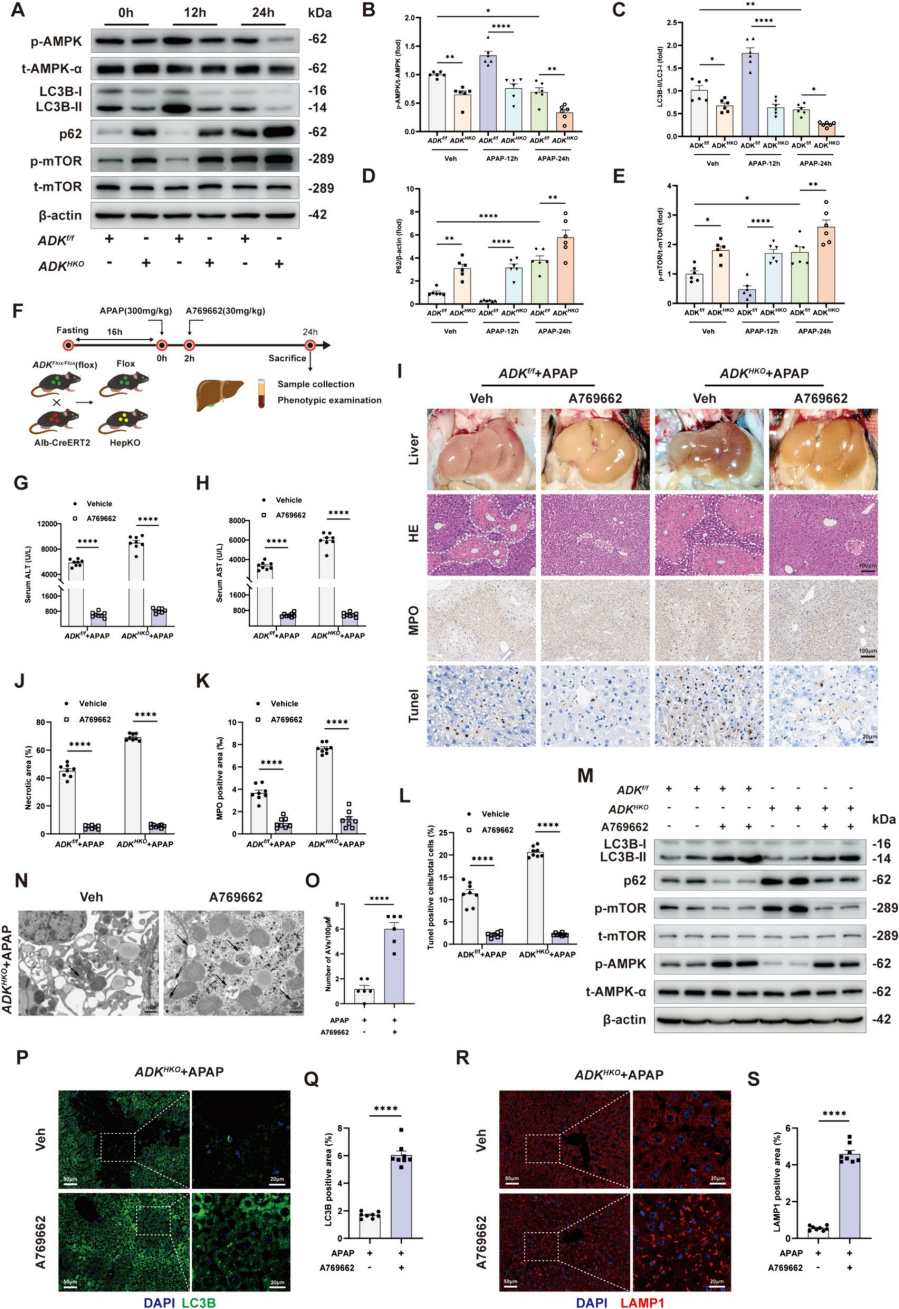
AMPK inactivation contributes to impaired autophagy in hepatic ADK-deficient livers. (**A**-**E**) Representative immunoblots and quantification of *ADK*^*f/f*^ and *ADK*^*HKO*^ mice at different time points after APAP challenge (n = 6 per group). In **F**-**S**, *ADK*^*f/f*^ and *ADK*^*HKO*^ mice were treated with A769662 (30 mg/kg) after APAP challenge (300 mg/kg, 24 h). (**F**) Schematic diagram of APAP and A769662 administration. (**G**-**H**) Serum ALT and AST levels (n = 8 per group). (**I**-**L**) Representative gross liver photographs and intrahepatic staining images with quantification of H&E (**J**), MPO (**K**), and TUNEL (**L**) staining (n = 8 per group). (**M**) Representative immunoblots of autophagy markers and AMPK (n = 6 per group). (**N**–**O**) Representative liver TEM image showing the quantification of AVs (black arrows, n = 6). (**P**-**S**) Representative immunofluorescence staining images and quantification of LC3B (**P**-**Q**, n = 8 per group) and LAMP1 (**R**-**S**, n = 8 per group) expression.^*^P < 0.05; ^**^P < 0.01; ^****^P < 0.0001; ns, nonsignificant (Student’s *t test*, one-way ANOVA with Tukey’s test, two-way ANOVA with Bonferroni’s multiple comparisons test, log-rank test)

AMPK is activated by AMP, which is produced from the ADK-mediated phosphorylation of adenosine, and AMPK activation facilitates the cellular autophagic process by inhibiting mTOR (Aymerich et al. 2006; Jia et al. 2020). AMPK phosphorylation and autophagy were both increased at 12 h after APAP treatment but attenuated at 24 h in mouse livers (Fig. 5A-D). In contrast, the phosphorylation of mTOR first decreased and then increased over time (Fig. 5A and E). In the context of hepatic ADK deficiency, both the activation of AMPK and autophagy were suppressed but the activation of mTOR was enhanced (Fig. 5A-E). RNA-sequencing analysis revealed that overexpressing ADK activated the AMPK-related pathway in APAP-injured mice (Supplemental Fig. 5A). Moreover, AMPK phosphorylation was increased in ADK-overexpressing livers (Supplemental Fig. 5B and C).

To elucidate whether AMPK activation contributes to the effect of ADK on APAP-induced ALI (300 mg/kg, 24 h), the AMPK activator A769662 was given to mice after APAP administration (Fig. 5F). AMPK activation decreased the serum ALT and AST levels (Fig. 5G and H). In addition, liver necrosis, neutrophil infiltration and cell death were alleviated by AMPK activation in APAP-treated *ADK*^*HKO*^ mice compared to *ADK*^*f/f*^ mice (Fig. 5I-L). Importantly, autophagy was activated by AMPK activation in APAP-injured livers from *ADK*^*HKO*^ mice (Fig. 5M-S; Supplemental Fig. 5D-G). To further determine whether AMPK activation protects against APAP-induced ALI through mTOR-mediated autophagy, the mTOR activator-MHY1485 was administered to mice before APAP and the AMPK activator (Supplemental Fig. 5H). We found that inhibiting autophagy by activating mTOR eliminated the protective effect of AMPK activation on APAP-induced liver necrosis, neutrophil infiltration and cell death in ADK^HKO^ mice (Supplemental Fig. 5I-N).

To confirm the critical role of AMPK in APAP-induced ALI (300 mg/kg, 24 h), another AMPK activator, AICAR, which activates AMPK through ADK, was administered to mice (Supplemental Fig. 6A). Interestingly, pretreatment with AICAR markedly decreased the serum ALT and AST levels in APAP-injured mice and prevented liver necrosis, neutrophil infiltration and cell death in APAP-injured livers, but the administration of AICAR after APAP treatment aggravated APAP-induced liver injury (Supplemental Fig. 6B-G). Moreover, pretreatment with AICAR activated autophagy, suppressed the phosphorylation of mTOR and increased the phosphorylation of AMPK, whereas the use of AICAR after APAP had the opposite effects (Supplemental Fig. 6H-K). The different effects of AICAR on APAP-induced liver injury might be explained by the decreased level of ADK in APAP-injured livers, and AICAR might exert AMPK-independent effects as an adenosine analog (Israeli et al. 2018). Further studies demonstrated that in *ADK*^*HKO*^ mice, pretreatment with AICAR failed to protect against APAP-induced ALI (Supplemental Fig. 6L-R). These results provide compelling evidence that AMPK activation is crucial for regulating autophagy mediated by ADK. Based on the different effects of AICAR on liver injury before and after APAP treatment, these results also indicate that in addition to AMPK, other signaling pathways may contribute to the aggravation of ADK on APAP-induced ALI.

**Fig. 6:**
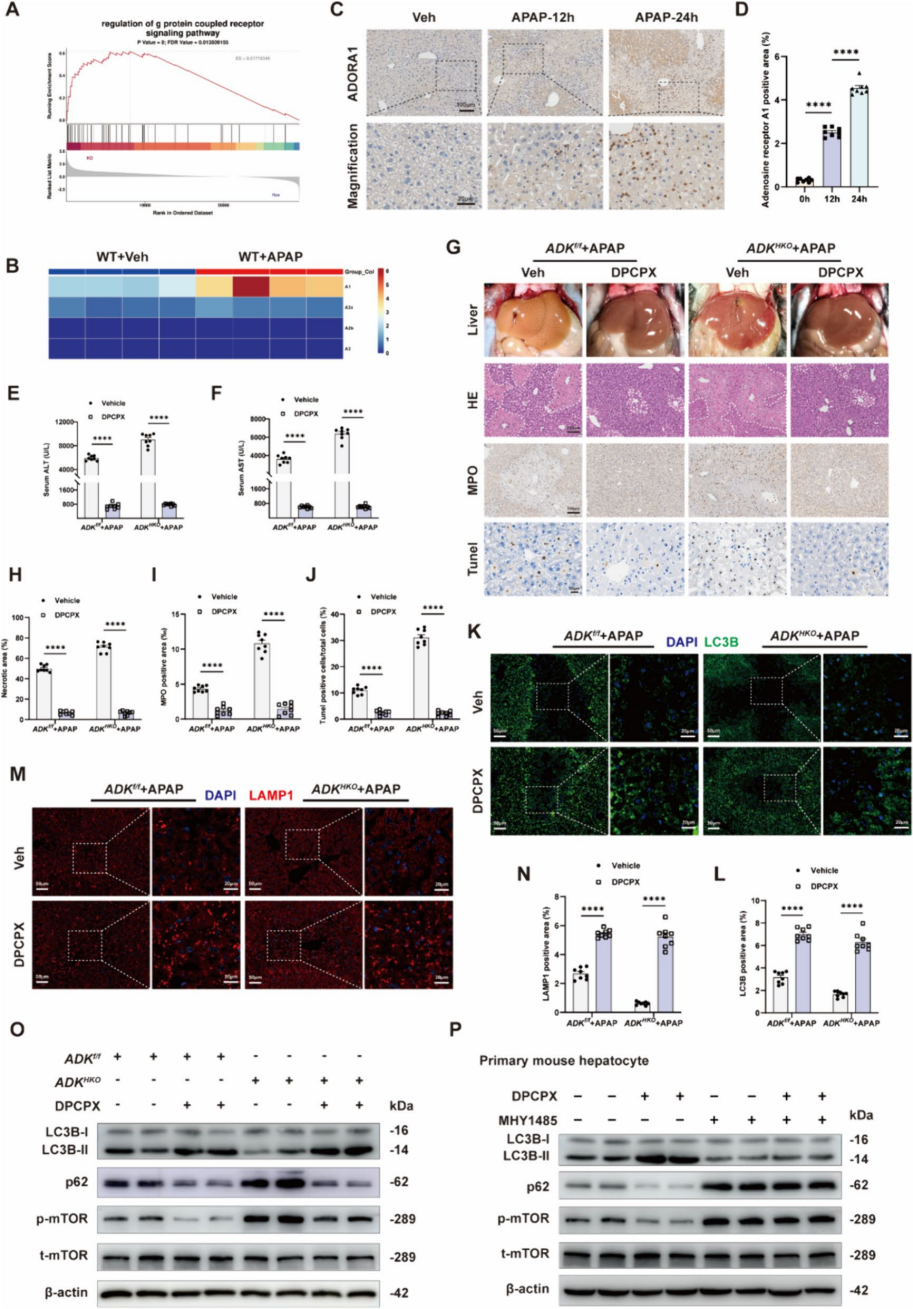
Activation of the adenosine receptor A1 contributes to APAP-induced liver injury. In **A-O**, mice were given intraperitoneal injection of 300 mg/kg APAP. (**A**) GSEA showing the enriched pathways related to G protein-coupled receptors and transmembrane signaling receptors based on the RNA-seq dataset of *ADK*^*f/f*^ and *ADK*^*HKO*^ mice treated with APAP for 24 h. (**B**) Heatmaps showing the intrahepatic expression profiles of four adenosine receptor subtypes in WT mice treated with APAP for 24 h based on the RNA-seq dataset. (both n = 4). (**C**-**D**) Representative immunohistochemical images and quantification of adenosine receptor A1 expression (n = 8 per group). In E-O, *ADK*^*f/f*^ and *ADK*^*HKO*^ mice were treated with DPCPX (0.5 mg/kg) after APAP challenge. (**E**–**F**) Serum ALT and AST levels (n = 8 per group). (**G**-**J**) Representative gross liver photographs and intrahepatic staining images with quantification of H&E (**H**), MPO (**I**), and TUNEL staining (**J**) (n = 8 per group). (**K**-**N**) Representative immunofluorescence staining images and quantification of LC3B (K-L, n = 8 per group) and LAMP1 (M–N, n = 8 per group) expression. (**O**) Representative immunoblots showing autophagy markers (n = 6 per group). In **P**, primary mouse hepatocytes from WT mice were treated with or without DPCPX (0.5 μM) or MHY1485 (10 μM) administration before APAP (10 mM) challenge. (**P**) Representative immunoblots showing autophagy markers (n = 6 per group). ^****^P < 0.0001 (one-way ANOVA with Tukey’s test, two-way ANOVA with Bonferroni’s multiple comparisons test)

### The activation of ADORA1 contributes to APAP-induced ALI

ADK deficiency can affect signaling pathways by increasing extracellular adenosine levels and activating adenosine receptors (Wang et al. 2021). To determine other possible pathways through which ADK affects APAP-induced ALI (300 mg/kg, 24 h), KEGG enrichment analysis was performed. Transmembrane signaling receptor activity was upregulated in APAP-injured *ADK*^*HKO*^ livers (Supplemental Fig. 7A). GSEA using the RNA-sequencing data demonstrated that the G protein-coupled receptor pathway was upregulated in APAP-injured *ADK*^*HKO*^ livers (Fig. 6A). Thus, we speculated that activation of adenosine receptors mediates the effect of ADK on APAP-induced liver injury. RNA sequencing analysis revealed that among the mRNAs of the four adenosine receptor subtypes, only the mRNA expression of ADORA1 was increased in APAP-injured mouse livers (Fig. 6B). ADORA1 was increased in sections of APAP-injured livers compared with control livers (Fig. 6C and D). Inhibiting the ADORA1 receptor with the antagonist DPCPX decreased the serum ALT and AST levels in APAP-injured *ADK*^*HKO*^ mice and alleviated APAP-induced ALI by reducing liver necrosis, neutrophil infiltration and cell death in both *ADK*^*HKO*^ mice and *ADK*^*f/f*^ mice (Fig. 6E-J). Moreover, inhibiting ADORA1 increased autophagy and suppressed the phosphorylation of mTOR in APAP-injured livers (Fig. 6K-O, Supplemental Fig. 7B-D). In vitro, these effects of ADORA1 inhibition were prevented by mTOR activation (Fig. 6P, Supplemental Fig. 7E-G).

**Fig. 7:**
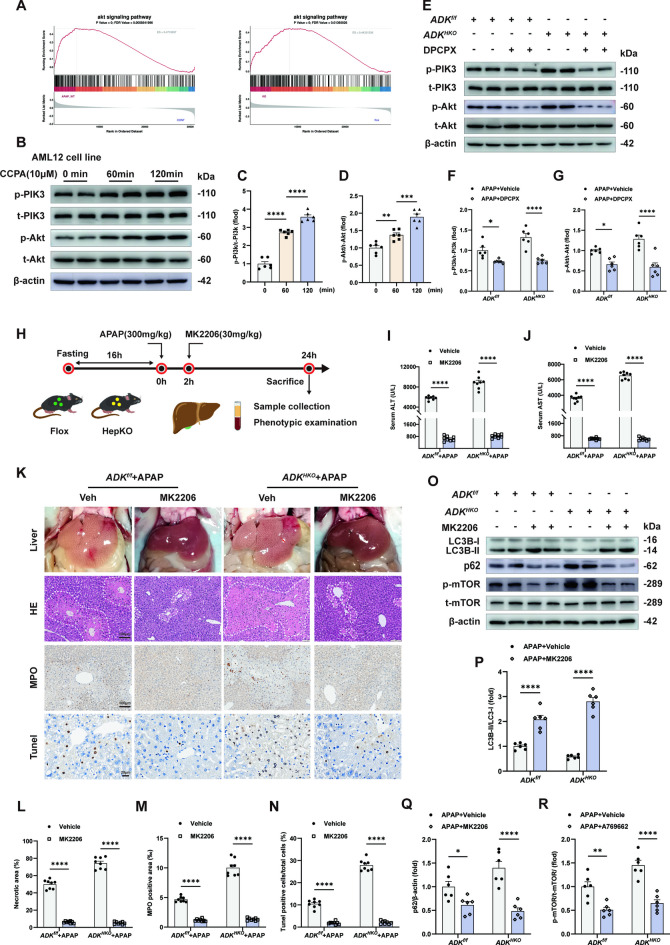
Adenosine receptor A1-dependent Akt activation promotes the phosphorylation of mTOR and suppresses autophagy in APAP-injured livers. (**A**) GSEA showing the enriched pathways related to Akt based on the RNA-seq dataset in WT and *ADK*^*HKO*^ mice treated with APAP (300 mg/kg) for 24 h. In B-D, AML12 cells were treated with CCPA (10 μM) at different time points. (**B**-**D**) Representative immunoblots with quantification of p-PI3K (**C**, n = 6 per group) and p-Akt expression (**D**, n = 6 per group). In **E–G**, *ADK*^*f/f*^ and *ADK*^*HKO*^ mice were treated with DPCPX (0.5 mg/kg) 2 h after APAP challenge (300 mg/kg). (**E**–**G**) Representative immunoblots showing the quantification of p-PI3K (**F**, n = 6 per group) and p-Akt expression (**G**, n = 6 per group). In **H**-**R**, *ADK*^*f/f*^ and *ADK*^*HKO*^ mice were treated with MK2206 (30 mg/kg) after APAP challenge. (**H**) Schematic diagram of APAP and MK2206 administration. (**I**-**J**) Serum ALT and AST levels (n = 8 per group). (**K**-**M**) Representative gross liver photographs and intrahepatic staining images with quantification of H&E (**L**), MPO (**M**), and TUNEL (**N**) staining (n = 8 per group). (**O**-**R**) Representative immunoblots showing the quantification of autophagy markers (n = 6 per group). ^*^P < 0.05; ^**^P < 0.01; ^***^P < 0.001; ^****^P < 0.0001; ns, nonsignificant (one-way ANOVA with Tukey’s test, two-way ANOVA with Bonferroni’s multiple comparisons test)

Proxyphylline is used as a cardiac stimulant, vasodilator and bronchodilator and is also an ADORA1 antagonist. To further confirm the role of ADORA1 inhibition in APAP-induced ALI, proxyphylline was administered after the mice were injured by APAP (Supplemental Fig. 7H). Proxyphylline treatment decreased the serum ALT and AST levels in APAP-injured mice (Supplemental Fig. 7I and J). In addition, liver necrosis, neutrophil infiltration and cell death were decreased by treating APAP-injured livers with proxyphylline (Supplemental Fig. 7K-N).

### ADORA1-dependent Akt activation promotes the phosphorylation of mTOR and suppresses autophagy in APAP-injured livers

KEGG enrichment analysis showed upregulation of the PI3K-Akt pathway in APAP-injured *ADK*^*HKO*^ livers (Supplemental Fig. 7A). GSEA analysis of the RNA-sequencing data indicated activation of the Akt pathway in APAP-injured livers from both wild type and *ADK*^*HKO*^ mice (Fig. 7A). In vitro, activating ADORA1 induced significant activation of the PI3K-Akt pathway (Fig. 7B-D, Supplemental Fig. 8A-C). The phosphorylation of Akt was significantly greater in APAP-injured *ADK*^*HKO*^ livers than in *ADK*^*f/f*^ livers, but inhibiting ADORA1 suppressed Akt phosphorylation and eliminated the ADK deficiency-induced increase in its phosphorylation (Fig. 7E-G). Akt inhibition prevented the aggravation due to ADK deficiency on APAP-induced ALI, which was indicated by the decreases in serum ALT and AST levels, liver necrosis, neutrophil infiltration and cell death (Fig. 7H-N). Moreover, autophagy was enhanced in APAP-injured *ADK*^*HKO*^ livers and this change was accompanied by decreased mTOR activation in response to Akt inhibition (Fig. 7O-R). Importantly, mTOR activation negated the protective effects of Akt inhibition on APAP-related ALI in *ADK*^*HKO*^ mice (Supplemental Fig. 8D-J). These findings suggest that the activation of ADORA1 exacerbates APAP-induced ALI by impairing autophagy through the Akt/mTOR pathway in *ADK*^*HKO*^ mice.

**Fig. 8:**
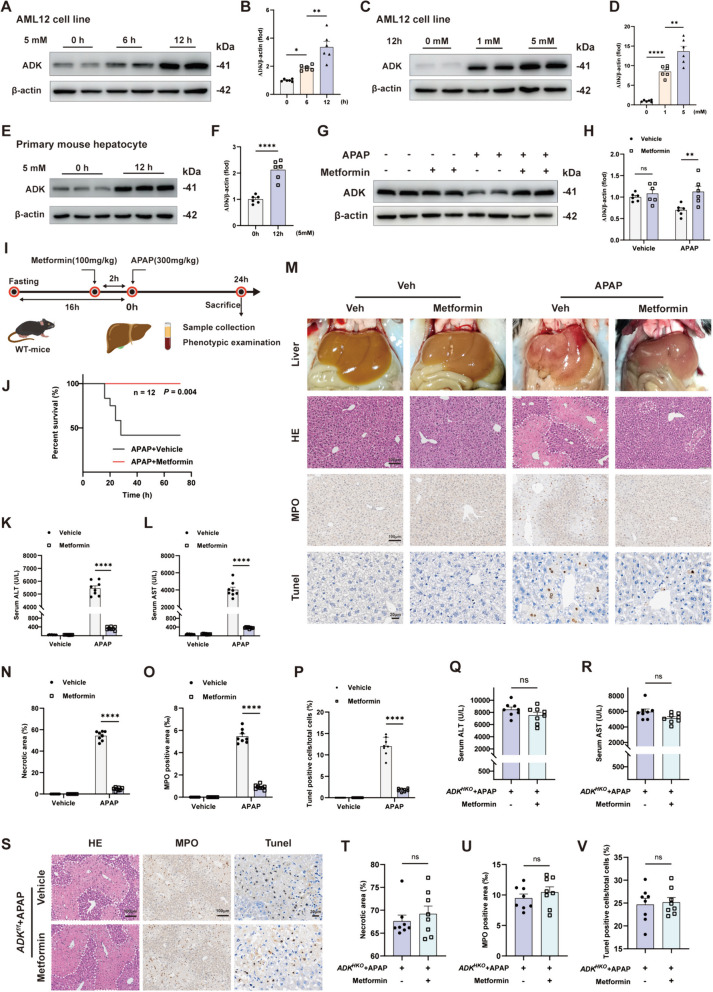
Metformin prevents APAP-induced acute liver injury by upregulating ADK. In **A**-**B**, AML12 cells were treated with metformin (5 mM) at different time points. Representative immunoblots (**A**) and quantification (**B**) of ADK expression (n = 6 per group). In **C-D**, AML12 cells were treated with different concentrations of metformin for 12 h. Representative immunoblots (**C**) and quantification (**D**) of ADK expression (n = 6 per group). In **E–F**, primary mouse hepatocytes were treated with 5 mM metformin for 12 h. Representative immunoblots (**E**) and quantification (**F**) of ADK expression (n = 6 per group). In **G**-**P**, WT mice were treated with metformin (100 mg/kg) before APAP challenge (300 mg/kg, 24 h). (**G**-**H**) Representative immunoblots showing the quantification of ADK expression (n = 6 per group). (**J**) Survival curves of WT mice treated with a lethal dose of APAP (650 mg/kg) in the absence or presence of metformin (both n = 12). (**K**-**L**) Serum ALT and AST levels (n = 8 per group). (**M**-**P**) Representative gross liver photographs and intrahepatic staining images with quantification of H&E (**N**), MPO (**O**), and TUNEL (**P**) staining (n = 8 per group). In **Q**–**V**, *ADK*^*HKO*^ mice were treated with metformin (100 mg/kg) before APAP challenge (300 mg/kg, 24 h). (**Q**-**R**) Serum ALT and AST levels (n = 8 per group). (**S**-**V**) Representative gross liver photographs and intrahepatic staining images with quantification of H&E (**T**), MPO (**U**), and TUNEL (**V**) staining (n = 8 per group). ^*^P < 0.05; ^**^P < 0.01; ^****^P < 0.0001; ns, nonsignificant (Student’s *t test*, one-way ANOVA with Tukey’s test; two-way ANOVA with Bonferroni’s multiple comparisons test, log-rank test)

### Metformin prevents APAP-induced ALI by upregulating ADK

As both ADK and metformin promote DNA methylation by regulating the level of S-adenosylhomocysteine (SAH), we speculate that metformin might affect the expression of ADK (Cuyas et al. 2018; Wahba et al. 2021). Fortunately, we observed that metformin elevated ADK expression in a manner dependent on both time and concentration (Fig. 8A-F). In APAP-injured livers (300 mg/kg, 24 h), 100 mg/kg of metformin can effectively reverse the downregulation of ADK expression. (Fig. 8G-H). Interestingly, metformin protected mice from death when a lethal dose (650 mg/kg) of APAP was administered (Fig. 8J). Moreover, metformin significantly protected against APAP-induced ALI, as indicated by decreases in ALT and AST levels, liver necrosis, neutrophil infiltration and cell death (Fig. 8K-P). To further validate that metformin exerts protective effects against APAP-induced ALI by increasing ADK expression, we administered metformin pretreatment (100 mg/kg) to APAP-induced *ADK*^*HKO*^ mice and found that the protective effects of metformin against APAP-related liver injury were completely abolished (Fig. 8Q-V).

## Discussion

The mechanisms underlying drug-induced ALI remain a research focus. In this study, we observed decreased ADK expression in APAP-injured mouse livers. Hepatic ADK deficiency aggravated APAP-related ALI while ADK overexpressing protected against ALI by regulating hepatic autophagy. The status of mTOR played a central role in the regulation of autophagy by ADK. As ADK phosphorylates adenosine to generate AMP, determining both the extracellular and intracellular levels of adenosine, we investigated the role of the AMPK and adenosine receptor pathways (Boison 2013). We found that both the AMPK and the ADORA1-Akt signaling pathways contributed to the effects of ADK on mTOR-dependent autophagy during APAP-induced ALI. Notably, metformin was proven to protect against drug-induced ALI by upregulating ADK, indicating that metformin might be useful for treating ALI in clinical practice.

Great efforts have been devoted to revealing the underlying mechanisms of ALI, especially APAP hepatotoxicity, and several important signaling pathways, including those involved in autophagy, inflammation, endoplasmic reticulum stress, programmed cell death, oxidative stress, and liver repair, have been identified in APAP-induced liver injury (Yan et al. 2018; Yoon et al. 2016).

Autophagy is a conserved self-degradative process in which abnormal proteins are removed and damaged organelles are cleared (Glick et al. 2010). Although autophagy is activated in the liver at the early stage of exposure to APAP, this activation is not sufficient to prevent APAP hepatotoxicity (Chao et al. 2018; Ni et al. 2012). In APAP-injured livers, autophagy helps clear APAP-protein adducts and subsequently prevents mitochondrial damage (McGill et al. 2013; Shan et al. 2018). Thus, enhancing the activation of autophagy protects against APAP-induced ALI(Chao et al. 2018; Ni et al. 2012). AMPK is a highly conserved cellular energy sensor that is activated when reduced ATP production results in relative increases in AMP or ADP (Jia et al. 2020; Kim et al. 2011). AMPK activation triggers autophagy through a dual mechanism involving direct activation of ULK1 and suppression of mTOR activation, which downregulates autophagy by inhibiting the phosphorylation of ULK1 and ULK2 (Mihaylova and Shaw 2011). AMPK was activated in the early stage of APAP injury, but both autophagy and AMPK were significantly suppressed 24 h after APAP overdose in the liver. Interestingly, the activation of autophagy and AMPK in the early stage was abolished by ADK deficiency in APAP-injured livers. Moreover, pretreatment with AICAR protected against APAP hepatotoxicity, but pretreatment with AICAR had no beneficial effect on preventing APAP injury in ADK-deficient livers. AICAR is converted by ADK to ZMP, which binds to AMPK and activates it directly; thus, AMPK activation plays a very important role in the effects of ADK on ALI (Dolinar et al. 2018).

Surprisingly, AICAR administration after APAP treatment worsened APAP-induced liver injury. As AICAR is also an adenosine analog, the potential roles of adenosine-related pathways have emerged. Adenosine is a nucleoside that primarily exerts its effects by activating its responding membrane receptors, adenosine receptors A1, A2A, A2B and A3 (Guieu et al. 2020; Sun et al. 2022). The level of adenosine increases in the extracellular space in response to metabolic stress and cell damage (Hasko et al. 2008). A previous study reported that during acute liver injury, the content of adenosine is elevated (Zhan et al. 2014). We found that the ADK level is reduced in APAP-injured livers. Based on the critical role of ADK in the metabolism of adenosine, the content of adenosine should also increase in APAP-injured livers. Thus, activation of the adenosine receptor should have an effect on APAP-induced liver injury. Although activation of the adenosine receptor A2B facilitates liver recovery after APAP injury, we found that only the expression of ADORA1 was increased in APAP-injured livers (Duan et al. 2022). Blockade of ADORA1 using an antagonist or genetic knockout protects against ethanol-induced fatty liver or alpha-naphthyl isothiocyanate-induced intrahepatic cholestatic liver injury (Peng et al. 2009; Yang et al. 2013). In the present study, blocking ADORA1 significantly alleviated APAP-induced liver injury and prevented aggravation of ADK deficiency on APAP-induced ALI. Proxyphylline, a selective ADORA1 antagonist, also protects against APAP-induced liver injury.

Another important finding is the upregulation of ADK by metformin. Metformin is the first-line medication for type 2 diabetes mellitus but the mechanism underlying its therapeutic action is still not fully understood (Foretz et al. 2023). SAH is generated from the conversion of S-adenosylmethionine (SAM) which is the substrate of DNA methyltransferases (DNMTs) (Wahba et al. 2021). Metformin promotes global DNA methylation by decreasing SAH levels, which has inhibitory effects on SAM-dependent DNMTs (Cuyas et al. 2018). Interestingly, ADK determines the intracellular and extracellular levels of adenosine, which is produced from the hydrolysis of SAH (Wang et al. 2021). Thus, it is reasonable to speculate the potential relationship between metformin and ADK. This finding provide insight into a new way to increase ADK expression. In addition, the changes in ADK levels might explain the beneficial effects of metformin on cancer, age-related diseases and inflammatory diseases which should be further investigated in the future.

In conclusion, ADK prevents drug-induced ALI by activating autophagy through the AMPK-mTOR and ADORA1-Akt-mTOR pathways. Metformin might be a potential medication for treating ALI by upregulating ADK.

## Supplementary Information

Below is the link to the electronic supplementary material.

Supplementary file1 (PDF 1894 KB)

## Data Availability

The datasets used and/or analyzed during the current study are available from the corresponding author upon reasonable request.
