# Supplementary material for: Adenosine kinase protects against acetaminophen-induced acute liver injury by activating autophagy in hepatocytes
Source: Cell Biol Toxicol. 2024 Jul 27;40(1):59. doi: 10.1007/s10565-024-09906-0 (PMC11281981; doi:10.1007/s10565-024-09906-0)
Supplement: Supplementary file 1 — Supplementary file1 (PDF 1894 KB) [file 10565_2024_9906_MOESM1_ESM.pdf]

Supplementary Materials

Adenosine kinase protects against acetaminophen-induced acute liver injury by activating autophagy in hepatocytes

Chuanxin Zhang, [1,2,3,4] Xuehao Liu, [1,2,3,4] Xilong Liu, [1,2,3,4] Rui Hua, [1,2,3,4], Han Liu, [1,2,3,4] Jiaxin Ma[1,2,3,4] Dan Zou, [1,2,3,4] Guangmei Wang, [1,2,3,4] Qiuhuan Yuan, [1,2,3,4] Bailu Wang, [5] Shujian Wei, [1,2,3,4*] Yuguo Chen, [1,2,3,4*]

[*]To whom correspondence should be addressed:

Email: chen919085@sdu.edu.cn or weishujian@sdu.edu.cn; No.107, Wen Hua Xi Road, Qilu Hospital of Shandong University, Jinan, Shandong, 250012, P.R. China.

Supplementary Figures and Figure legends

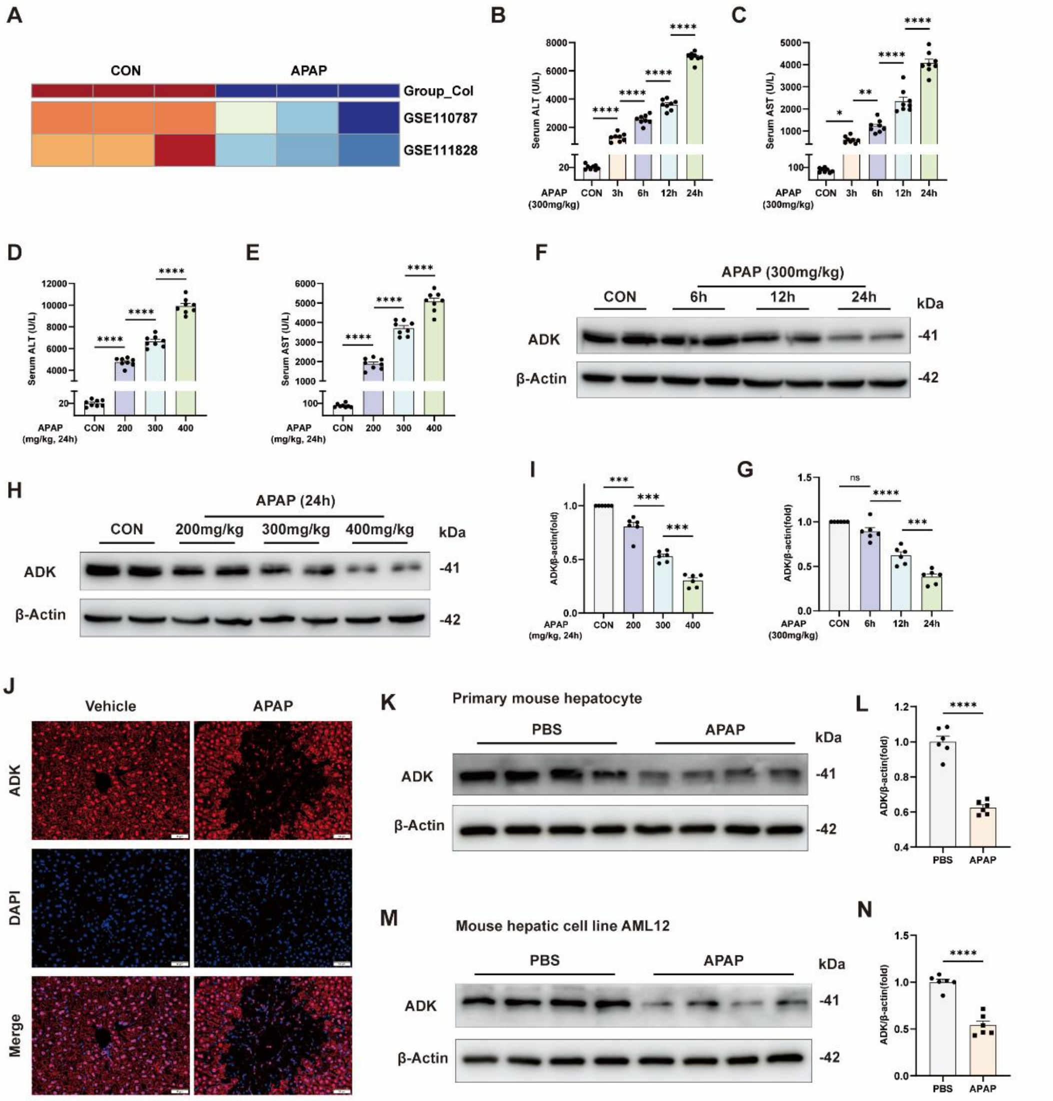

Supplementary Fig. 1 The expression of ADK is downregulated in APAP-injured hepatocytes. (A) The APAP-induced ALI datasets GSE110787 and GSE111828 were obtained from the GEO database, and the mRNA level of ADK was displayed using a heatmap. In B-C, F-G, WT mice were intraperitoneally injected with vehicle or APAP solution (300 mg/kg). Blood and liver tissue was obtained at 0, 3, 6, 12, and 24 h (n= 8 per group). In D-E, H-I, WT mice were intraperitoneally injected with saline or various doses of APAP solution (200, 300, and 400 mg/kg). Then, they were sacrificed at 24 h. (B-E) Serum ALT and AST. (F-I) Representative immunoblots with quantification of ADK (n=6). (J) WT mice were intraperitoneally injected with vehicle or APAP solution (300 mg/kg, 24h). Representative immunofluorescence staining images of ADK. (K-L) Primary mouse hepatocytes from WT mice were treated with or without APAP (10 mM, 24 h) (n=6 per group). Representative immunoblots and quantification of ADK. (M-N) AML12 cells were treated with or without APAP (10 mM, 24 h) (n=6 per group).

Representative immunoblots and quantification of ADK. $^{*}P$ <0.05; $^{**}P$ <0.01; $^{***}P$ <0.001; $^{****}P$ <0.0001; ns, nonsignificant (Student's *t test*, one-way ANOVA with Tukey's test).

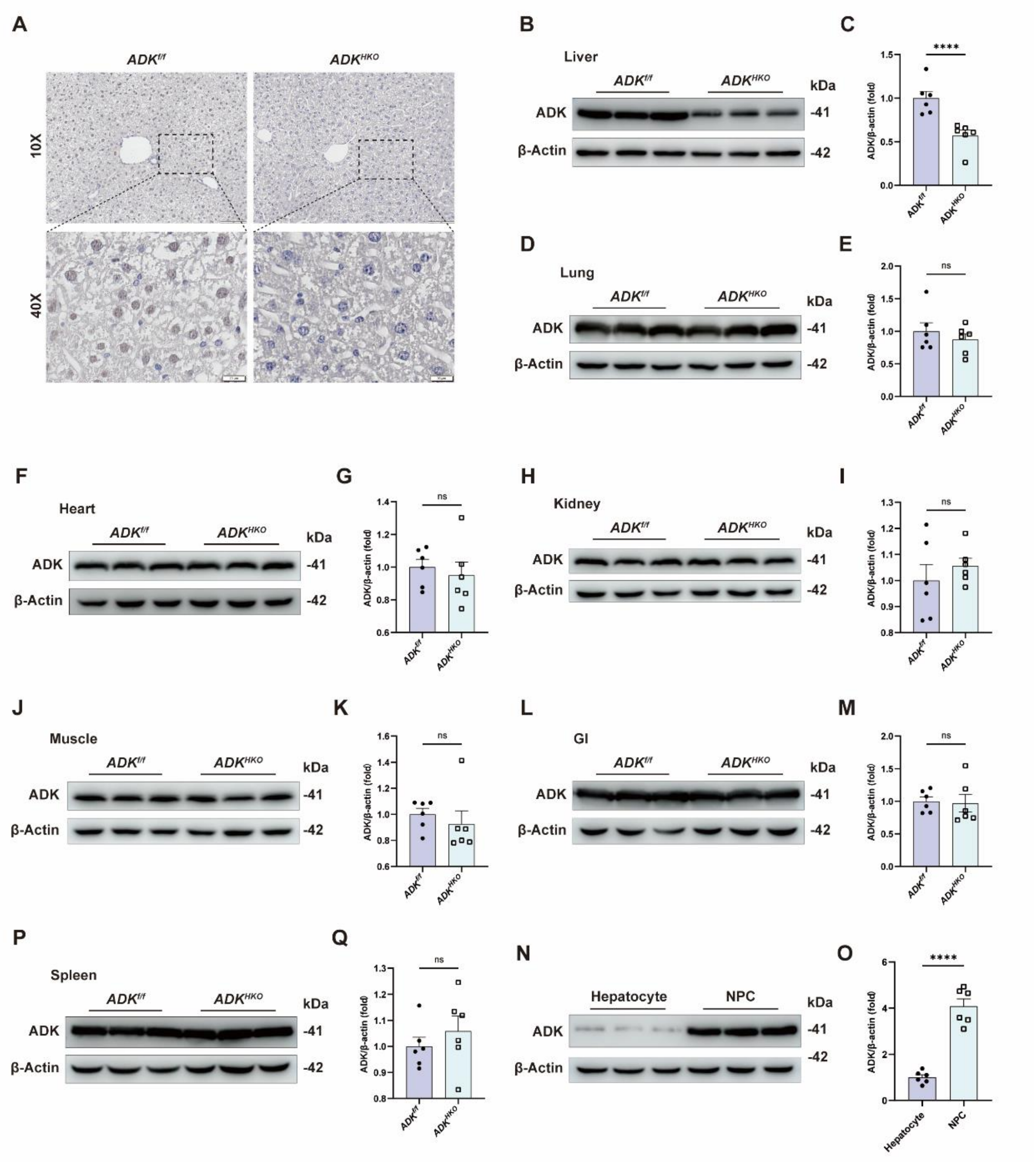

Supplementary Fig. 2 ADK conditional knockout in hepatocytes. (A) Representative immunohistochemical images of ADK from the liver tissue of $ADK^{f/f}$ and ADK^{HKO} mice. (B-Q) Representative immunoblots and quantification of ADK from various tissues of $ADK^{f/f}$ and ADK^{HKO} mice (n=6 per group). In N-O, primary mouse hepatocytes and NPC were extracted from the liver tissue of ADK^{HKO} mice. Representative immunoblots and quantification of ADK (n=6 per group). $^{****}P$ <0.0001 (Student's t test). $^{**}P$ <0.01; $^{****}P$ <0.0001; ns, nonsignificant (Student's *t test*, one-way ANOVA with Tukey's test).

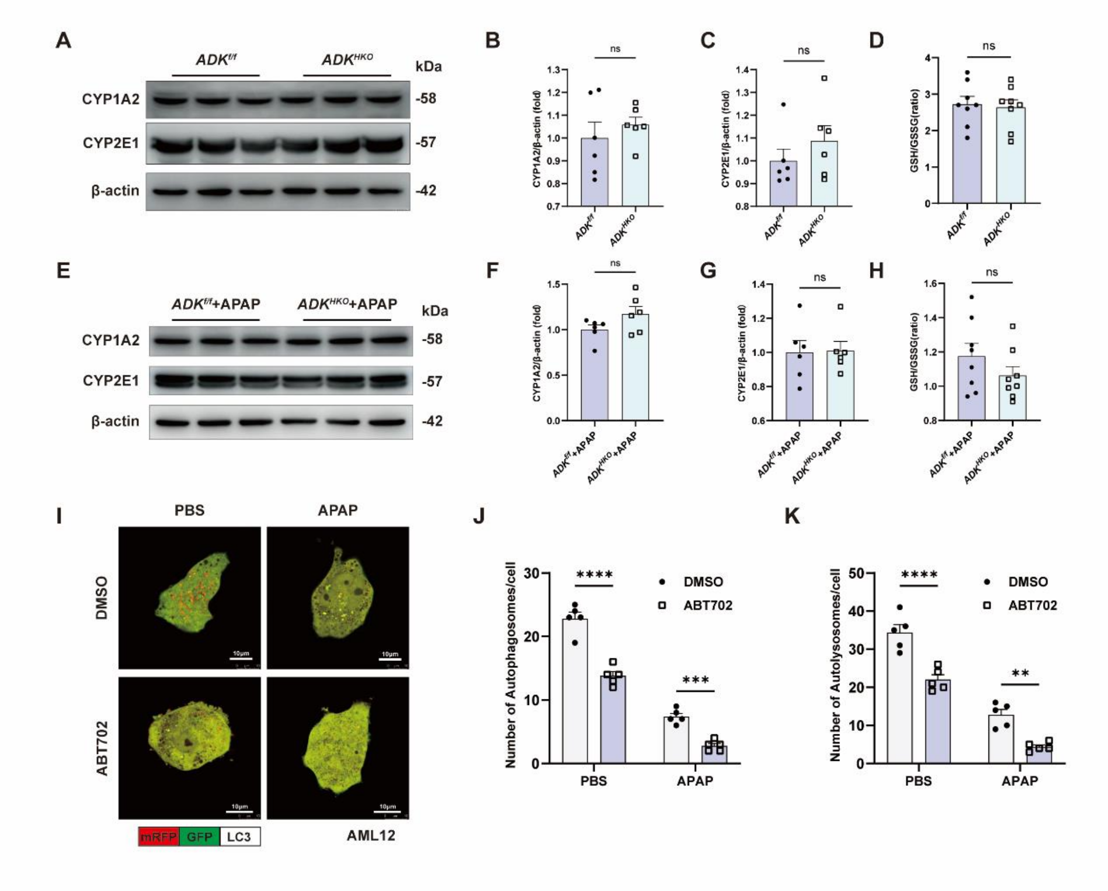

Supplementary Fig. 3 Autophagy was impaired by ADK knockout in APAP-injured mouse livers. (A-C) Representative immunoblots and quantification of CYP1A2 (B) and CYP2E1 (C) in *ADK*$^{f/f}$ and *ADK*^{HKO} mice (n=6 per group). (D) Hepatic levels of GSH/GSSG ratio in *ADK*$^{f/f}$ and *ADK*^{HKO} mice (n=6 per group). In E-G, *ADK*$^{f/f}$ and *ADK*^{HKO} mice were intraperitoneally injected with APAP solution (300 mg/kg) for 24 h. Representative immunoblots and quantification of CYP1A2 (F) and CYP2E1 (G) in APAP-induced *ADK*$^{f/f}$ and *ADK*^{HKO} mice (n=6 per group). (H) Hepatic levels of GSH/GSSG ratio in APAP-induced *ADK*$^{f/f}$ and *ADK*^{HKO} mice (n=6 per group). In I-K, AML12 cells were treated with or without the preadministration of ABT702 (1 μM) before the APAP (10 mM) challenge. Representative confocal images (I) of the mRFP-GFP-LC3 reporter in AML12 cells and quantification (J-K) of autophagic flux. $^{**}P$ <0.01; $^{***}P$ <0.001; $^{****}P$ <0.0001; ns, nonsignificant (Student's t test, two-way ANOVA with Bonferroni's multiple comparisons test).

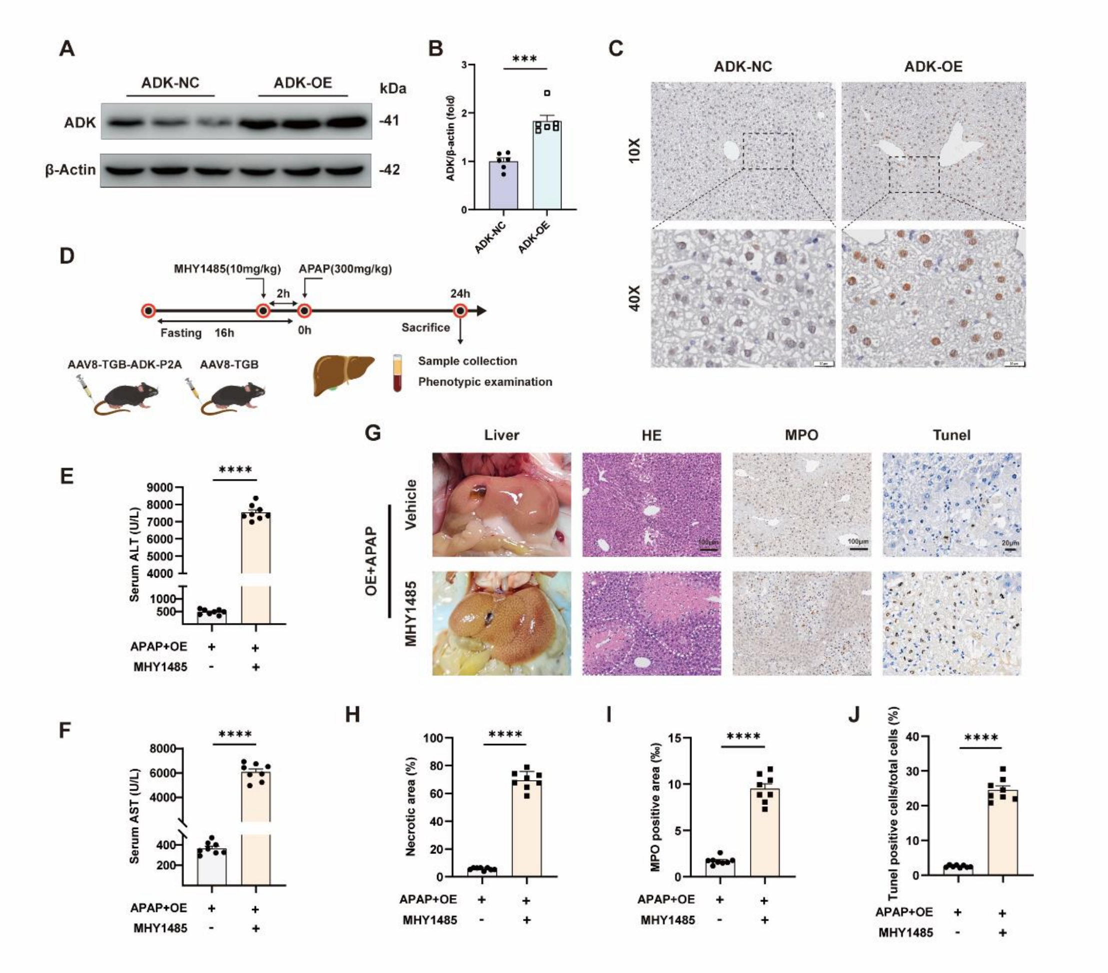

Supplementary Fig. 4 ADK overexpression alleviates APAP-induced mouse hepatotoxicity through the mTOR pathway. In A-J, WT mice were injected with AAV8-TBG-ADK-P2A or AAV8-TBG adeno-associated virus 30 days before APAP challenge. (A) Representative immunoblots and quantification of ADK from the liver tissue of ADK-NC and ADK-OE mice (n=6 per group). (C) Representative immunohistochemical images of ADK from the liver tissue of ADK-NC and ADK-OE mice. In D-J, the ADK-OE mice were pretreated with MHY1485 (10 mg/kg) before the APAP challenge (n=8 per group). (E-F) Serum ALT and AST. (G-J) Representative gross liver photographs and intrahepatic staining with quantification of H&E (H), MPO (I), and TUNEL (J). $^{***}P$ <0.001; $^{****}P$ <0.0001 (Student's *t test*).

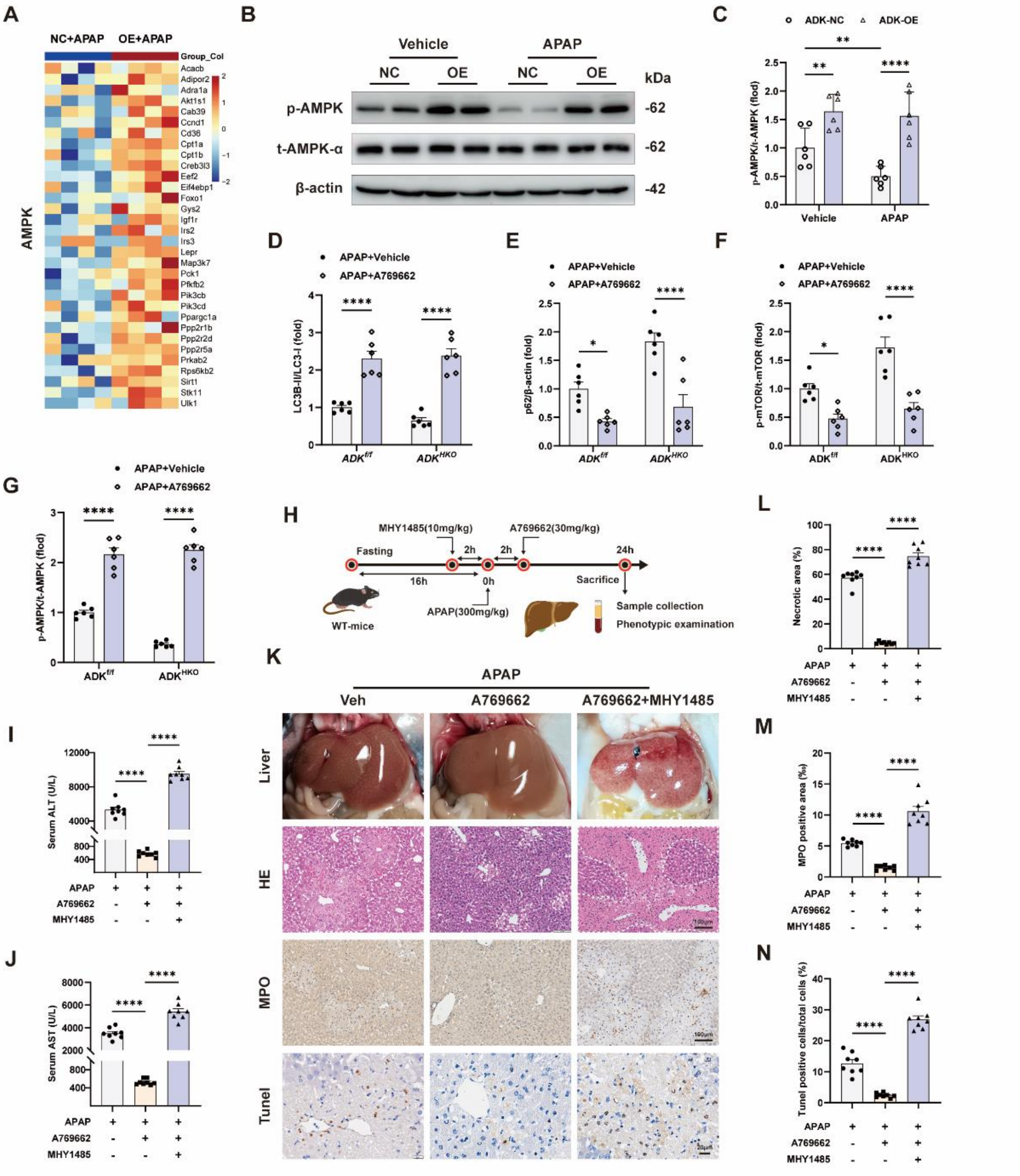

Supplementary Fig. 5 ADK overexpression alleviates APAP-induced mouse hepatotoxicity through AMPK-mTOR signaling axis. In A-C, WT mice were injected with AAV8-TBG-ADK-P2A or AAV8-TBG adeno-associated virus 30 days before the APAP challenge. (A) Heatmaps showing the intrahepatic expression profile of genes related to autophagy and AMPK based on the RNA-seq dataset (n=4). (B-C) Representative immunoblots and quantification of p-AMPK (n=6 per group). In D-G, $ADK^{f/f}$ and ADK^{HKO} mice were treated with A769662 (30 mg/kg) after the APAP challenge. Quantification of representative immunoblots of autophagy (D-F) and p-AMPK (G). In H-N, WT mice were pretreated with MHY1485 (10 mg/kg) before inducing DILI and then treated with or without A769662 (30 mg/kg) after the APAP challenge (n=8 per group). (I-J) Serum ALT and AST. (K-N) Representative gross liver photographs and intrahepatic staining with quantification of H&E (L), MPO (M), and TUNEL (N). $^{*}P$ <0.05; $^{**}P$ <0.01; $^{***}P$ <0.001; $^{****}P$ <0.0001 (Student's *t test*, two-way

ANOVA with Bonferroni’s multiple comparisons test).

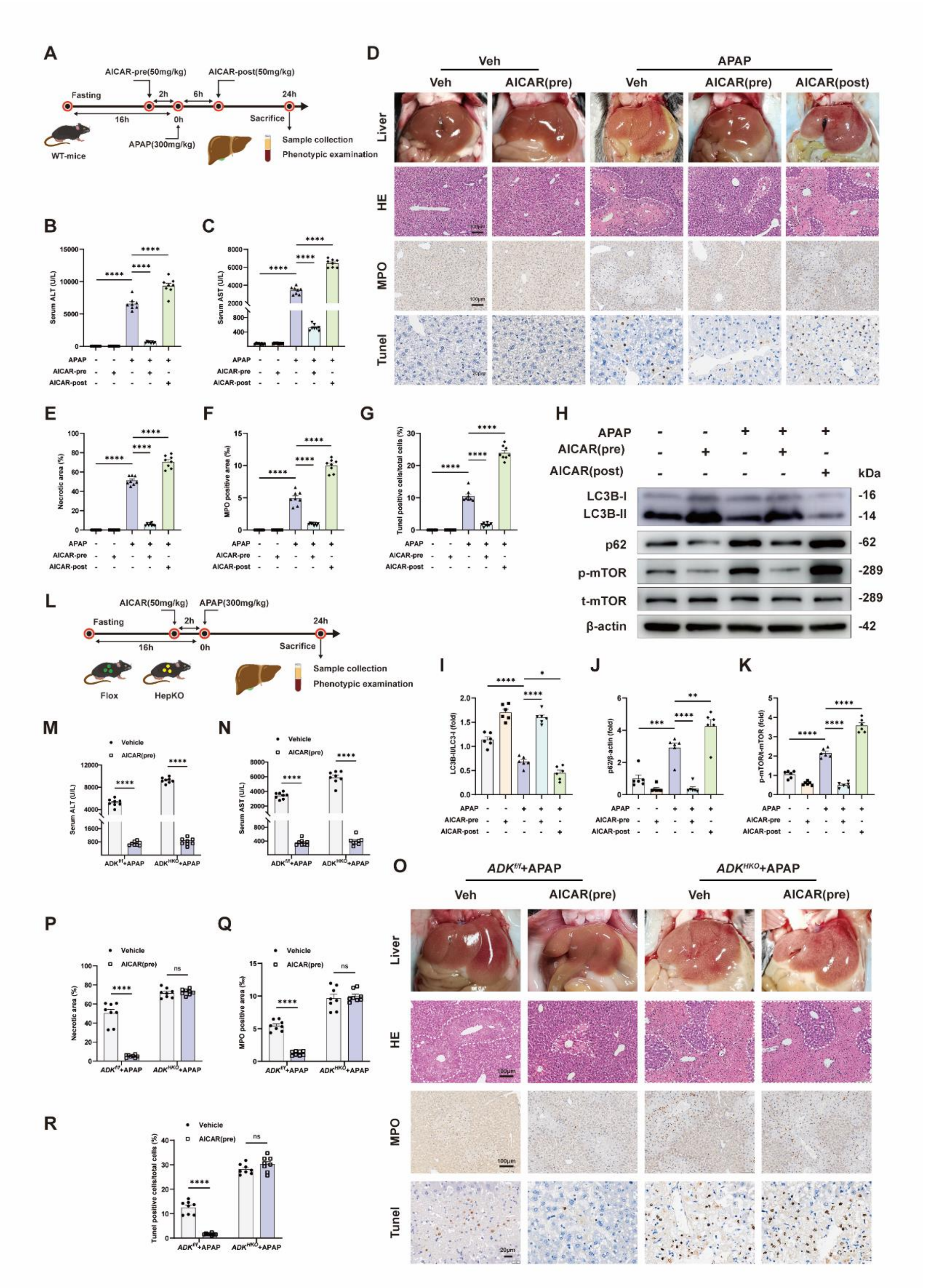

Supplementary Fig. 6 AICAR post-treatment aggravates APAP-induced liver injury. In A–K, WT mice were treated with AICAR (50 mg/kg) before or after the APAP challenge. (A) Schematic diagram of APAP and AICAR administration. (B-C) Serum ALT and AST (n=8 per group). (D-G) Representative gross liver photographs and intrahepatic staining with quantification of H&E (E), MPO (F), and TUNEL (G)

(n=8 per group). (H-K) Representative immunoblots with quantification of autophagy (n=6 per group). In L-R, the *ADK*$^{f/f}$ and *ADK*^{HKO} mice were treated with AICAR (50 mg/kg) before the APAP challenge (n=8 per group). (R) Schematic diagram of APAP and AICAR administration. (M-N) Serum ALT and AST. (O-R) Representative gross liver photographs and intrahepatic staining with quantification of H&E (P), MPO (Q), and TUNEL (R). $^{*}P$ <0.05; $^{**}P$ <0.01; $^{***}P$ <0.001; $^{****}P$ <0.0001 (one-way ANOVA with Tukey's test, two-way ANOVA with Bonferroni's multiple comparisons test). APAP, acetaminophen; WT, wild type.

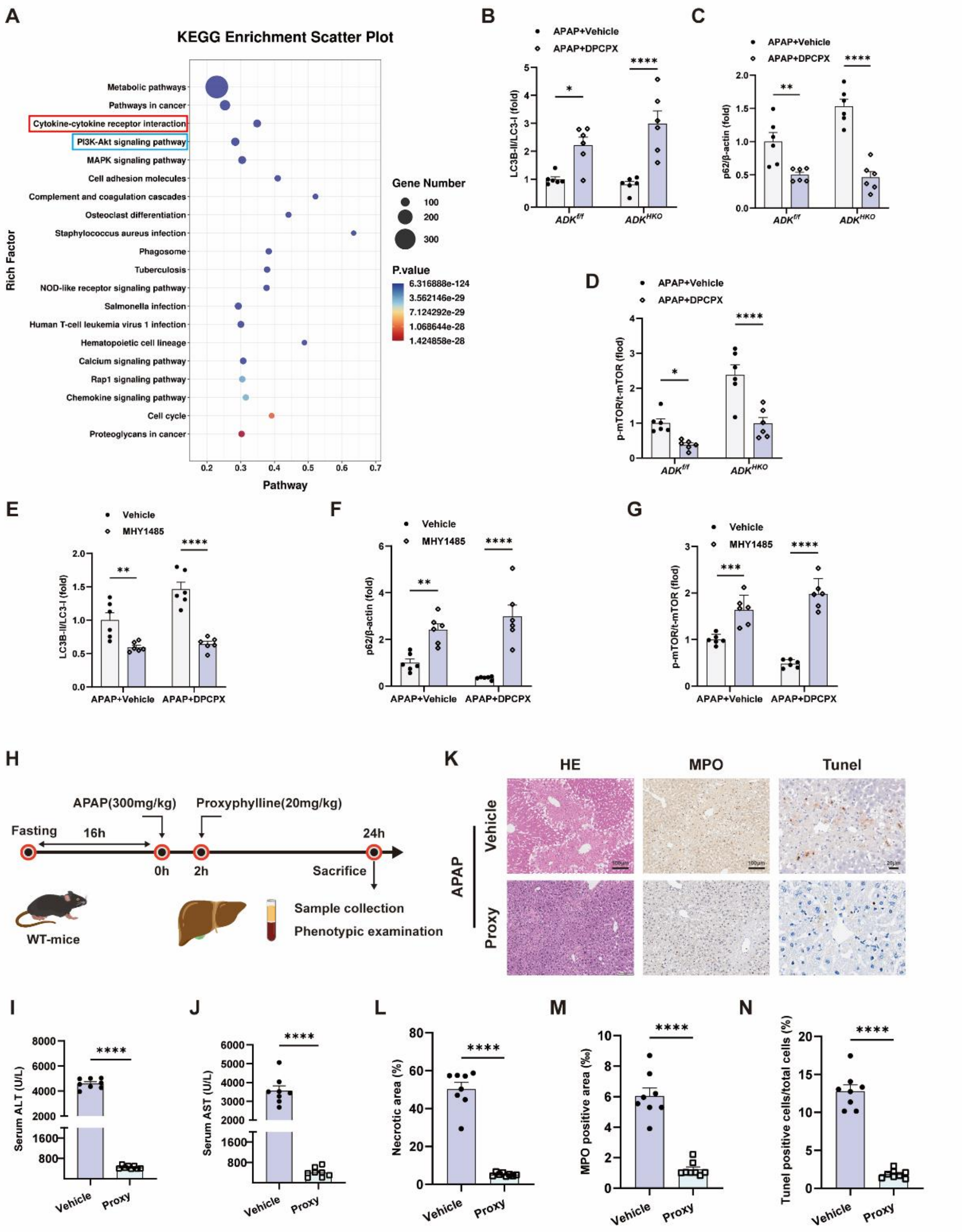

Supplementary Fig. 7 The inhibition of adenosine receptor A1 can alleviate APAP-induced liver injury. (A) KEGG pathway-enrichment analysis of differentially expressed pairs in *ADK*$^{f/f}$ and *ADK*^{HKO} mice treated with APAP. The top 20 pathways (Fisher's exact test) are presented. In B-D, the *ADK*$^{f/f}$ and *ADK*^{HKO} mice were treated with DPCPX (0.5 mg/kg) after the APAP challenge. (B-D) Quantification of representative immunoblots of autophagy (n=6 per group). In E-G, primary mouse hepatocytes from WT mice were treated with or without the preadministration of DPCPX (0.5 μM) or MHY1485 (10 μM) before the APAP (10 mM) challenge. (E-G) Quantification of representative immunoblots of autophagy (n=6 per group). In H-N, WT mice were treated with proxyphylline after the APAP challenge. Blood and liver

tissue of mice were obtained at 24 h. (H) Schematic diagram of APAP and proxyphylline administration. (I-J) Serum ALT and AST. (K-N) Representative intrahepatic staining with quantification of H&E (L), MPO (M), and TUNEL (N). $^{*}P$ <0.05; $^{**}P$ <0.01; $^{****}P$ <0.0001 (Student's *t test*, two-way ANOVA with Bonferroni's multiple comparisons test).

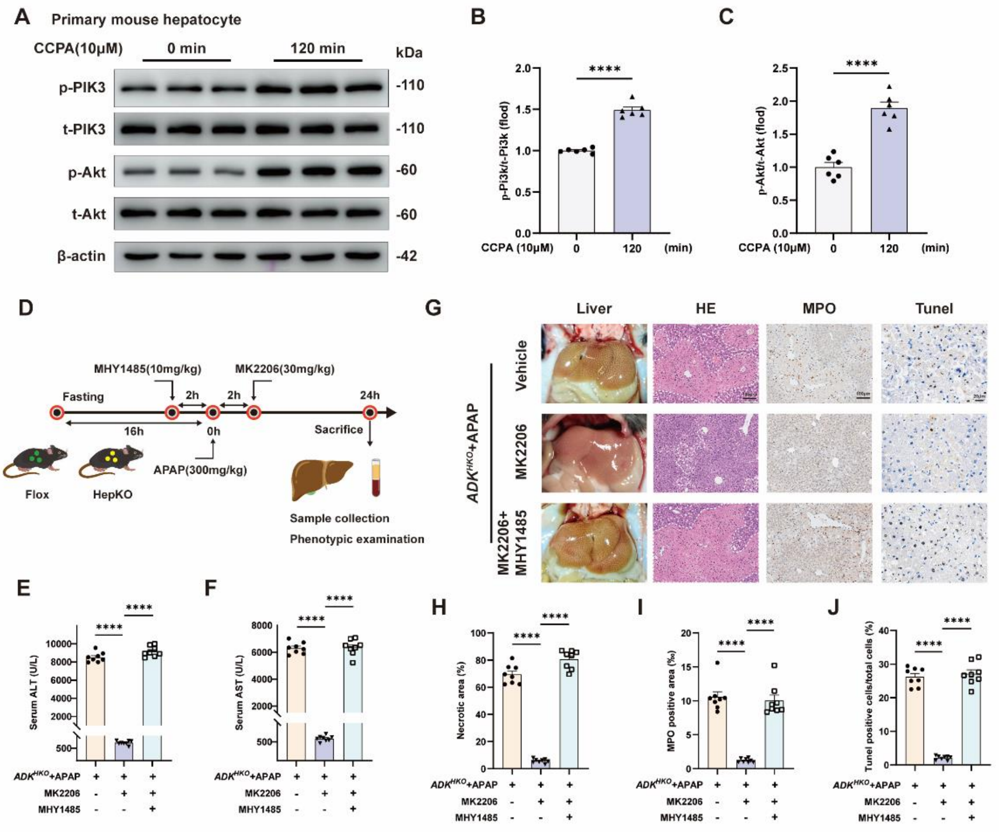

Supplementary Fig. 8 ADK knockdown exacerbates APAP-induced mouse hepatotoxicity via the AKT-mTOR signaling axis. In A-C, primary mouse hepatocytes were treated with CCPA (10 μM) at different time points. Representative immunoblots with quantification of p-PI3K (C, n=6 per group) and p-Akt expression (D, n=6 per group). In D-J, $ADK^{f/f}$ and ADK^{HKO} mice were pretreated with MHY1485 (10 mg/kg) before inducing DILI and then treated with or without MK2206 (30 mg/kg) after the APAP challenge (n=8 per group). (E-F) Serum ALT and AST. (G-J) Representative gross liver photographs and intrahepatic staining with quantification of H&E (H), MPO (I), and TUNEL (J). $^{****}P$ <0.0001 (one-way ANOVA with Tukey's test).

Supplemental Table. 1 Demographic features of enrolled DILI patients.

Group	Gender	Age (Years)	Causative drug	ALT	AST
Control-1	M	51	—	61	15
Control-2	F	50	—	29	52
Control-3	M	53	—	17	25
Control -4	M	44	—	50	55
DILI-1	M	48	APAP	661	1473
DILI-2	M	30	APAP	988	2612
DILI-3	M	54	APAP	2043	1608
DILI-4	M	40	APAP	1511	769

Abbreviations: F, Female; M, Male; ALT, alanine transaminase; AST, glutamic oxaloacetic transaminase

Supplemental Table. 2 The detailed information of in vivo administration of small-molecule activators and inhibitors.

Name	Function	Dose and frequency	Time point	Administration mode
**Rapamycin** (Selleck; s1039)	Autophagy activator	4 mg/kg once	2 h after	intraperitoneal
**A769662** (Selleck; s2697)	AMPK activator	30 mg/kg once	2 h after	intraperitoneal
**AICAR** (Selleck; s1802)	AMPK activator	50 mg/kg once	2 h before or 6 h after	intraperitoneal
**DPCPX** (Selleck; E1310)	ADORA1 inhibitor	0.5 mg/kg	2 h after	intraperitoneal
**MHY1485** (Selleck; s7811)	mTOR activator	10 mg/kg once	2 h before	intraperitoneal
**MK2206** (Selleck; s1078)	Akt1/2/3 inhibitor	30 mg/kg once	2 h after	intraperitoneal
**Metformin** (Selleck; s1950)	—	100 mg/kg once	2 h before	intraperitoneal
**Proxyphylline** (Selleck; s4932)	ADORA1 inhibitor	20 mg/kg once	2 h after	intraperitoneal

Abbreviations: AMPK, AMP-activated protein kinase; ADORA1, adenosine receptor A1.

Supplemental Table 3. Antibody information

Antibody	Source	Application	MW (kDa)
**Rabbit polyclonal anti-ADK**	Abcam; ab227087	IHC (1:200)<br>WB (1:1000)	41
**Mouse monoclonal anti-β-actin**	Proteintech; 66009-1-lg	WB (1:10000)	42
**Rabbit polyclonal anti-MPO**	Proteintech; 22225-1-AP	IHC (1:200)	-
**Rabbit HNF-4-alpha**	Abclonal; A20865	IF (1:200)	-
**Rabbit monoclonal anti-LC3B**	Abcam; ab192890	WB (1:2000)<br>IF (1:200)	14, 16
**Rabbit monoclonal anti-p62**	Abcam; ab109012	WB (1:10000)	62
**Rabbit monoclonal anti-mTOR**	Abcam; ab32028	WB (1:2000)	289
**Rabbit monoclonal anti-p-mTOR (S2448)**	Abcam; ab109268	WB (1:2000)	289
**Rabbit monoclonal anti-LAMP1**	Cell Signaling Technology; #73294	IF (1:100)	-
**Rabbit monoclonal anti-p-AMPKα (Thr172)**	Cell Signaling Technology; #2535	WB (1:1000)	62
**Rabbit polyclonal anti-AMPKα**	Cell Signaling Technology; #2532	WB (1:1000)	62
**Rabbit polyclonal anti-Adenosine Receptor A1**	Proteintech; 20332-1-AP	IHC (1:200)	-
**Rabbit monoclonal anti-Akt**	Cell Signaling Technology; #4685	WB (1:1000)	60
**Rabbit monoclonal anti-p-Akt (Ser473)**	Cell Signaling Technology; #4060	WB (1:1000)	60
**Rabbit monoclonal anti-PI3K**	Cell Signaling Technology; #4249	WB (1:1000)	110
**Rabbit polyclonal anti-p-PI3K (Tyr458/Tyr199)**	Affinity; AF3242	WB (1:1000)	110
**Rabbit polyclonal anti-CYP2E1**	Proteintech; 19937-1-AP	WB (1:2000)	57
**Rabbit polyclonal anti-CYP1A2**	Proteintech; 19936-1-AP	WB (1:1000)	58

Abbreviations: MW, molecular weight; IHC, immunohistochemistry; WB, western blot; IF, immunofluorescence.
